# Isolation of bioactive compounds from medicinal plants used in traditional medicine: Rautandiol B, a potential lead compound against *Plasmodium falciparum*

**DOI:** 10.1186/s12906-021-03406-y

**Published:** 2021-09-13

**Authors:** Christiana J. Dawurung, Minh T. H. Nguyen, Jutharat Pengon, Kanchana Dokladda, Ratchanu Bunyong, Roonglawan Rattanajak, Sumalee Kamchonwongpaisan, Phuong T. M. Nguyen, Stephen G. Pyne

**Affiliations:** 1 School of Chemistry and Molecular Bioscience, Faculty of Science Medicine and Health, University of Wollongong, Wollongong, NSW 2522 Australia; 2 Department of Veterinary Physiology, Biochemistry and Pharmacology University of Jos, Jos Plateau State, Nigeria; 3 Department of Life Science, University of Science and Technology of Hanoi, Vietnam Academy of Science and Technology, 18 Hoang Quoc Viet, Cau Giay, Hanoi, Vietnam; 4 National Center for Genetic Engineering and Biotechnology, National Science and Technology Development Agency, Pathum Thani, 12120 Thailand; 5 Department of Plant Biochemistry, Institute of Biotechnology, Vietnam Academy of Science and Technology, 18 Hoang Quoc Viet, Cau Giay, Hannoi, Vietnam

**Keywords:** Bioactivity, Phytochemicals, Traditional medicine, Crude extracts

## Abstract

**Background:**

*Neorautanenia mitis*, *Hydnora abyssinica*, and *Senna surattensis* are medicinal plants with a variety of traditional uses. In this study, we sought to isolate the bioactive compounds responsible for some of these activities, and to uncover their other potential medicinal properties.

**Methods:**

The DCM and ethanol extracts of the roots of *N*. *mitis* and *H. abyssinica*, and the leaves of *S. surattensis* were prepared and their phytochemical components were isolated and purified using chromatographic methods. These extracts and their pure phytochemical components were evaluated in in-vitro models for their inhibitory activities against *Plasmodium falciparum*, *Trypanosoma brucei rhodesiense*, *Mycobacterium tuberculosis,* α-amylase (AA), and α-glucosidase (AG).

**Results:**

Rautandiol B had significant inhibitory activities against two strains of *Plasmodium falciparum* showing a high safety ratio (SR) and IC_50_ values of 0.40 ± 0.07 μM (SR - 108) and 0.74 ± 0.29 μM (SR - 133) against TM4/8.2 and K1CB1, respectively. While (−)-2-isopentenyl-3-hydroxy-8-9-methylenedioxypterocarpan showed the highest inhibitory activity against *T. brucei rhodesiense* with an IC_50_ value of 4.87 ± 0.49 μM (SR > 5.83). All crude extracts showed inhibitory activities against AA and AG, with three of the most active phytochemical components; rautandiol A, catechin, and dolineon, having only modest activities against AG with IC_50_ values of 0.28 mM, 0.36 mM and 0.66 mM, respectively.

**Conclusion:**

These studies have led to the identification of lead compounds with potential for future drug development, including Rautandiol B, as a potential lead compound against *Plasmodium falciparum.* The relatively higher inhibitory activities of the crude extracts against AG and AA over their isolated components could be due to the synergistic effects between their phytochemical components. These crude extracts could potentially serve as alternative inhibitors of AG and AA and as therapeutics for diabetes.

**Supplementary Information:**

The online version contains supplementary material available at 10.1186/s12906-021-03406-y.

## Background

Medicinal plants are useful in the treatment of many ailments and diseases among rural dwellers, indigenous users, traditional medicine (TM) practitioners, and livestock owners in many African countries. The traditional knowledge of medicinal plants if harnessed, can give insights into the vital role that medicinal plants play in drug development [1–3]. Often, a single medicinal plant can have multiple uses, and sometimes different parts of the same plant may be used for the treatment of more than one disease condition. Other times, the same plant could be used as an ingredient in herbal preparations for a synergistic effect [1, 4, 5]. This is made possible due to the range of phytochemicals that are present in medicinal plants along with their diversities of bioactivities. *Neorautanenia mitis* (A. Rich) Verdc. (Fabacae)*, Hydnora abyssinica* A. Braun (Hydnoraceae), and *Senna surattensis* (Burm. f.) H. Irwin and Barneby (Fabaceae), were selected based on their promising preliminary screening results, they have shown various bioactivities and are traditionally used for the treatments of many disease conditions. The roots of *N. mitis,* are used for the treatment of bilharzia, syphilis, diarrhea, skin infection, dysmenorrhea and neuropsychiatric conditions. They are also used as an anticonvulsant, anti-malarial, fish poison, insecticide, and for killing bilharzias-carrying fresh water snails in many African countries [6–10]. The crude extracts and phytochemical constituents isolated from *N. mitis* have shown antidiarrheal [3, 11], acaricidal, insecticidal [12], antinocicetive, anti-inflammatory [9, 13], larvicidal, mosquitocidal [14], cytotoxicity [11, 15], and antimicrobial, activities [16]. *H. abyssinica*, is referred to as one of the strangest plants in the world, with its vegetative body consisting of only flowers, fruits and roots and has no leaves. It is not very common among botanists and plant scientists because it is rarely encountered [17–19]. However, it remains a popular and valuable medicinal plant among local users and TM practitioners, and is traded by traditional medicine vendors in local markets in South Africa, Mozambique and Nigeria [3, 19–23]. In some African countries including, Sudan, Kenya, South Africa, Malawi, Mozambique and Nigeria, it has been used for, the treatment of diarrhea, severe bacterial infections such as urinary tract infection, helminthiasis, internal wounds, piles, acne and dysentery, the expulsion of retained placenta and the treatment of throat and stomach aches [3, 17, 21, 24–27]. Extracts and constituents from *H. abyssinica* showed immunosuppressive [25], cytotoxic, antibacterial [17], antioxidant [28], molluscidal [29] and antidiarrheal activities [3].

*S.* surattensis is spread across tropical and subtropical countries, it is used as a food and as an ingredient in herbal mixtures. The roots are used for the treatment of gonorrhoea and snake bites, the leaves are used to treat dysentery and the flowers as a pugative [30, 31]. The crude extracts and phytochemical constituents from *S.* surattensis *have shown* antimicrobial [32, 33], antioxidant [33–35], antidiabetic [36, 37], antidiarrheal [3], hepatoprotective [35, 38], anthihyperlipidimic and antihyperglycemic activities [39].

In our continuous search for bioactive phytochemical constituents from medicinal plants, we evaluated the extracts and pure isolated phytochemicals from the three selected medicinal plants for their inhibitory activities against *Plasmodium falciparum*, *Trypanosoma brucei rhodesiense*, *Mycobacterium tuberculosis,* α-amylase (AA) and α-glucosidase (AG). These biological targets are associated with, malaria, human African trypanosome (HAT), tuberculosis and diabetes. The selections of these disease targets were based on the matching traditional uses of these plants and the need for newer drugs to aid in the combat against the growing drug resistance problems that are being encountered in the treatment of these conditions. Furthermore, malaria, HAT and tuberculosis are classified as re-emerging diseases [40, 41]. Malaria is a very important disease in Africa, it is caused by virulent *Plasmodium falciparum* which is transmitted through a bite of the female anopheles mosquito. It is endemic in most tropical countries of Asia, Africa and South America. A WHO report showed estimated deaths of 405,000 from 228 million infected cases in 2018 [42]. Malaria is both preventable and curable, and many anti-malaria drugs are available and control measures against the mosquito vector are also in place, however, resistance by the causative agent to each new class of drug has been reported, and this poses a grave challenge in combating this disease [43]. Therefore it is important to continue screening for new therapies and drug development leads, especially from alternative natural sources.

## Methods

These experiments were set up to isolate and characterized compounds from the selected medicinal plants, their extracts and pure compounds were evaluated against *P. falciparum, T. brucei rhodesiense*, *M. tuberculosis*, α-amylase (AA) and α-glucosidase (AG) in in-vitro models.

### General experimental procedures

The NMR data were recorded on Bruker Avance with Cryoprobe (500 MHz) or Bruker Ascend (400 MHz) NMR spectrometers in d_4_-methanol and deuterated chloroform (CDCl_3_) with tetramethylsilane (TMS) as internal standard. The elucidation of all chemical structures was aided by IR, MS, NMR (1D and 2D), MP, and optical rotation. FTIR Shimadzu IRAffinity-1 with MIRacle was used to measure IR spectra. MS were measured on a LCMS-2020 Shimadzu for ESI, while HRSIMS were taken on Thermo Scientific Electron Transfer Dissociated (ETD) Orbitrap Fusion FSN 10314–1. A Buchi M.560 melting point apparatus and a Jasco P-2000 polarimeter were used to measure melting points and specific rotations, respectively.

### Collection and identification of plants

All plants were collected from Kabwir Village in Kanke Local Government Area of Plateau State, Nigeria (Latitude 9^o^ 29′ 18.2′′ N and 9^o^ 29′ 30.0′′ E) in July 2016. They were submitted for identification and authentication to the Department of Plant Science and Technology University of Jos, Nigeria where voucher/reference numbers were assigned as follows, *N. mitis* (UJ16000246), *S. surattensis* (UJ16000248), and *H. abyssinica* (UJ16000248) [3].

### Water extraction

The aqueous extracts for this study were from the same batch of plant material as described in our previous study [3].

### Organic solvent extraction

The roots of *N. mitis,* were chopped into small pieces and dried in an oven at 45 °C for 2 days, while the leaves of *S. surattensis* were removed from the small stems and dried in a hot air oven at 45 °C for 2 days. The roots of *H. abyssinica* were cut into small pieces and dried in a hot air oven at 45 °C for 5 days. The dried plant materials were pulverized using a mortar and pestle. Measured amounts of the different plant materials (*N*. *mitis*-1000 g, *S. surattensis*- 405 g, *H. abyssinica*– 1000 g) were successively extracted with DCM and ethanol using a ratio of 1:4 plant material:solvent, for 72 h. The extracts were filtered through a sieve with a pore size of 150 μm, a cotton plug and then filter paper. Filtrates were evaporated and dried under a constant stream of air provided by a laboratory electric fan overnight, to obtain the dried crude DCM and ethanol extracts and their appearances and yields were recorded [44].

### Isolation of phytochemicals from *S. surattensis*

The phytochemical components of the ethanol extract (CEOH) from *S. surattensis* (10 g) were separated by flash column chromatography (CC) over silica gel with increasing gradient solvent polarity from MeOH/DCM (1.5:85) to 100% MeOH. A total of 12 fractions were obtained (F1-F12). Fraction F2 (369.2 mg) was purified by CC over Sephadex (LH-20) by elution with 100% MeOH to obtain 4 fractions (F2F1-F2F4). F2F3 was evaporated and identified as compound **1** (11.0 mg), while fraction F2F4 was identified as compound **2** (18.4 mg). Fractions F3-F4 (524.5 mg) were combined and purified by CC over Sephadex (LH-20) by elution with 100% MeOH to obtain 4 sub-fractions (F34f1-F34f4). Sub-fraction F34F3 (214 mg) was further purified by CC over Sephadex (LH-20) by elution with 100% MeOH to obtain 9 sub-fractions (F34F3f1-F34F3f9). Sub-fractions F34F3f6-F34F3f6 were combined and evaporated and identified as compound **3** (40.6 mg).

### Isolation of phytochemicals from *H. abyssinica*

About 50 g of the crude ethanol extract (KEOH) from *H. abyssinica* was partitioned with solvents from low polarity (hexanes) to high polarity (*n*-butanol). The extract was dissolved in a mixture of distilled water and MeOH (10:90) and extracted with 200 mL of hexanes in a separating funnel and allowed to settle before the hexanes portion was collected. This process was repeated three times (200 mL × 3) to obtain the hexane extract (4.7 g) after evaporation of the volatiles under reduced pressure. In a similar way, the aqueous MeOH solution was then extracted with DCM (200 mL × 3) to obtain the DCM extract (8.70 g). The same extraction procedure was repeated using ethyl acetate (200 mL × 3), acetone (200 mL × 3), and *n*-butanol (200 ml × 3) in that order, to obtain the ethyl acetate (833 mg), acetone (3.9 g) and *n*-butanol (1.8 g) extracts. About 2 g of the DCM extract was separated by CC over silica gel with MeOH/DCM (10:90) as eluent to obtain 9 fractions (F1-F9). Fraction F5 was evaporated and identified as compound **3** (197 mg), and fraction F6 as compound **4** (45.8 mg).

### Isolation of phytochemicals from *N. mitis*

A total of 14 g of the DCM crude extract (ABDCM) from *N. mitis* was separated by CC over silica gel using a gradient system from ethyl acetate (EtOAc)/hexanes (1:9) to 100% EtOAc to yield 40 fractions; these were combined based on their similarities by TLC and NMR analysis to afford 12 fractions (F1-F12). Compounds **5**–**24** were isolated through repeated CC, PTLC and CC over Sephadex LH-20 as previously described [11].

### Antimalaria assay against *P. falciparum* - TM4/8.2 and K1CB1

*Plasmodium falciparum* TM4/8.2 and K1CB1 strains were maintained in RPMI 1640 medium supplemented with 8% human serum, 2.5 mM HEPES and 2 g/L sodium bicarbonate. Parasite cultures were incubated at 37 °C in a 3% CO_2_ incubator. The crude extracts/pure compounds were tested against TM4/8.2 and K1CB1 *P. falciparum* in vitro by the modified [^3^H] hypoxanthine incorporation assay [45, 46]. Briefly, stock solutions of the crude extracts or pure compounds were prepared in DMSO. Various concentrations of the samples were incubated with malaria parasites with a final volume of 225 μL, 1% parasitemia, 1.5% hematocrit, 0.1% dimethylsulfoxide (DMSO) in 96-well plates. Plates were incubated for 16–18 h. Then, 25 μL [^3^H]-hypoxanthine solution was added and incubated for 20 h. The parasites were harvested onto a UniFilter-96 GF/B plate (PerkinElmer, USA). The filters in the plates were air-dried, and then 25 μL of liquid scintillation fluid (Microscint, Packard) was added. The radioactivity was then measured using a microplate scintillation counter (TopCount, Packard). The IC_50_ values of the crude extracts/ pure compounds were determined.

### Anti-trypanasomal assay (*T. brucei rhodesiense*)

*T. brucei rhodesiense* (STIB-900) was maintained in Minimal Essential Medium (MEM) with Earle’s salts supplemented with 3 g/L sodium bicarbonate, 4.5 g/L glucose, 25 mM HEPES, pH 7.3, 0.05 mM bathocuproinedisulfonic acid disodium salt, 1.5 mM L-cysteine, 1 mM hypoxanthine, 0.16 mM thymidine, 1 mM sodium pyruvate, 0.2 mM 2-mercaptoethanol, 1% MEM non-essential amino acid, and 15% heated fetal bovine serum, at 37 °C in a 5% CO_2_ incubator. To assay anti-Tbr activity, a modified reported protocol was used [47, 48]. Briefly, 2 × 10^4^ *T. brucei rhodesiense* parasites in 175 μL culture media were incubated with 25 μL of varying concentrations of each compound in a 96-well plate under the same culture conditions. Following 72 h incubation, 20 μL Alamar Blue (a resazurin solution) was added in each well. The mixture was further incubated for 3 h. The fluorescence signals were measured by a spectrofluorometer at ex530/em585 nm. The results were read as concentration of each compound that exhibit 50% growth inhibition (IC_50_) from the dose-response curve established from the fluorescence signals at each concentration of compounds.

### *Mycobacterium tuberculosis* assay

*Mycobacterium tuberculosis* H37Rv (ATCC 27294) were grown in Löwenstein–Jensen (LJ) medium agars (Biomedia Thailand cat. no. BMT B24420) in a biosafety level 3 facility at the Department of Microbiology, Faculty of Science, Mahidol University. Two–three loopfuls of each *M. tuberculosis* strain from the LJ medium were scratched and put into a tube containing 10 mL of Middlebrook 7H9 broth supplemented with 0.2% glycerol and 10% Middlebrook OADC. Clumps of colonies were dispersed using a loop. The bacterial suspensions were left undisturbed for 15 min before the upper parts were collected. Absorbance at OD_600_ was measured. Finally, bacterial suspensions were prepared to OD_600_ at 0.005. Anti-tuberculous activities were evaluated by the Microplate Alamar Blue Assays [49]. The assay was performed in a 96-well microplate. From stock 10 mg/mL in dimethyl sulfoxide, two-fold serial dilution of compounds/crude extracts (0.312–20 μg/mL) were prepared in Middlebrook 7H9 broth supplemented with 0.2% glycerol and 10% Middlebrook OADC in a 96-well plate to the volume of 100 μL. Then 100 μL of *M. tuberculosis* (OD_600_ of 0.005) was added to each well. Bacterial growth control and compounds/crude extracts control wells contained the medium with either bacteria or the test sample, respectively, while the medium control wells were without both. All were done in triplicate. The plate was incubated in a 37 °C incubator for 5 days. Then 20 μL of alamar blue and 12.5 μL of 20% Tween80 were added to one well of each control and the plate was further incubated for 24 h to test bacterial growth by observing color change. A blue color in the well was interpreted as no bacterial growth while a pink color represented proper bacterial growth. Therefore, if the well was still blue, the additional control wells were tested daily until the color changed to pink. Then the reagents were added to the entire plate and incubated for 24 h. Minimum inhibitory concentration (MIC) was then read from the lowest concentration of samples that prevented the change from blue to pink. Isoniazid and rifampin were employed as controls.

### Assay for α-glucosidase inhibition activity (AGI)

AGI values of extracts and pure phytochemicals were *quantitatively* determined in a 96-well plastic plate according to the method described by Nguyen et al. [50]. The enzyme was incubated with the samples dissolved in DMSO for 5 min before adding the substrate. The inhibition was measured spectrophotometrically in 20 mM sodium phosphate buffer pH 6.0 at 37 °C using 3.0 mM *p*-nitrophenyl α-D-glucopyranoside (Sigma) as the substrate and 0.25 units/mL of AG from *S. cerevisiae* (Sigma). The absorbance at 405 nm was measured using a microplate reader (BioTek ELx808 microplate reader, USA). Acarbose (5 mM) was used as the positive control.

### Assay for α-amylase inhibitory activity (AAI)

AAI values were determined using a microplate-based starch–iodine assay. Assay reactions were initiated by adding 40 μL of starch (Sigma S-2630) solution (2.0 g/L) and 40 μL of enzyme in 0.1 M phosphate buffer at pH 7.0 to microplate wells. The enzyme was incubated with the samples for 5 min before adding the substrate. To minimize evaporative loss during incubation, a plastic mat was used to cover the microplate. After 30 min of incubation at 50 °C, 20 μL of 1 M HCl was added to stop the enzymatic reaction, followed by the addition of 100 μL of iodine reagent (5 mM I_2_ and 5 mM KI). Following color development, the absorbance at 580 nm was measured using a microplate reader (Bio-TEK ELx808 microplate reader, USA) [51].

## Results

The DCM and ethanol extracts from *S. surratensis* were coded from its species name ‘cassia’ as CDCM and CEOH, respectively. While the DCM and ethanol extracts of *H. abyssinica* were coded from its local name ‘kaushe’ as KDCM and KEOH, respectively. And the DCM and ethanol extracts from *N. mitis* were coded from its local name ‘abargora’ as ABDCM and ABEOH, respectively. The color and appearances and percentage yields of the DCM and ethanol extracts from all three plants are reported in Table 1, while that of the water extracts (*S. surrattensis*-CAQ, *H. abyssinica*-KAQ and *N. mitis*-ABAQ) were described earlier [3]. Higher percentage yields of 11.6 and 10.3% were observed from the ethanol extracts of *S. surrattensis* and *H. abyssinica* respectively, compared to their DCM extracts. However, the ethanol extract of *N. mitis* had a lower yield of 1.0% while its DCM yield was 1.5%.

**Table 1:** Physical appearance and percentage yield of DCM and ethanol extracts from *S. surratensis*, *H. abyssinica* and *N. mitis*

Extract	Colour/appearance	Dry plant material (g)	Amount of extract (g)	Percentage yield (%)
CDCM	Green solid	405	11.0	2.7
CEOH	Green sticky solid	380	44.0	11.6
KDCM	Brick red solid	1000	10.0	1.0
KEOH	Crystal like dark brown solid	970	100.0	10.3
ABDCM	Dark brown solid	1000	15.0	1.5
ABOH	Dark brown solid	975	10.0	1.0

### Isolation of phytochemicals

One extract from each plant, which were CEOH from *S. surrattensis*, KEOH from *H. abyssinica* and ABDCM from *N. mitis*, was selected for phytochemical studies which involved the isolation and structural characterisation of the pure chemical constituents. This selection was based on their promising preliminary biological activities and TLC profiles.

### Phytochemicals from CEOH and KEOH

**Fig. 1:**
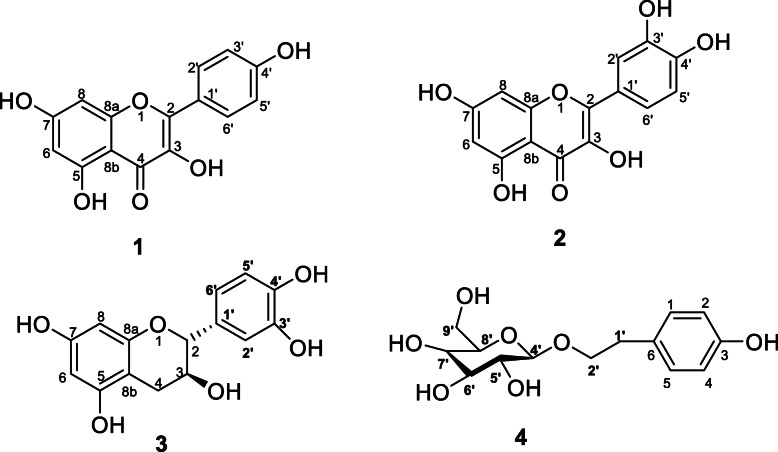
Compounds isolated from CEOH (**1**–**3**) and KEOH (**3** and **4**)

Three known compounds where isolated from CEOH, they were; kempherol (**1**) [52], quercetin (**2**) [38], and (+)-catechin (**3**), which was also isolated from KEOH [53] together with salidroside (**4**) [54] (Fig. 1).

### Phytochemicals from ABDCM

**Fig. 2:**
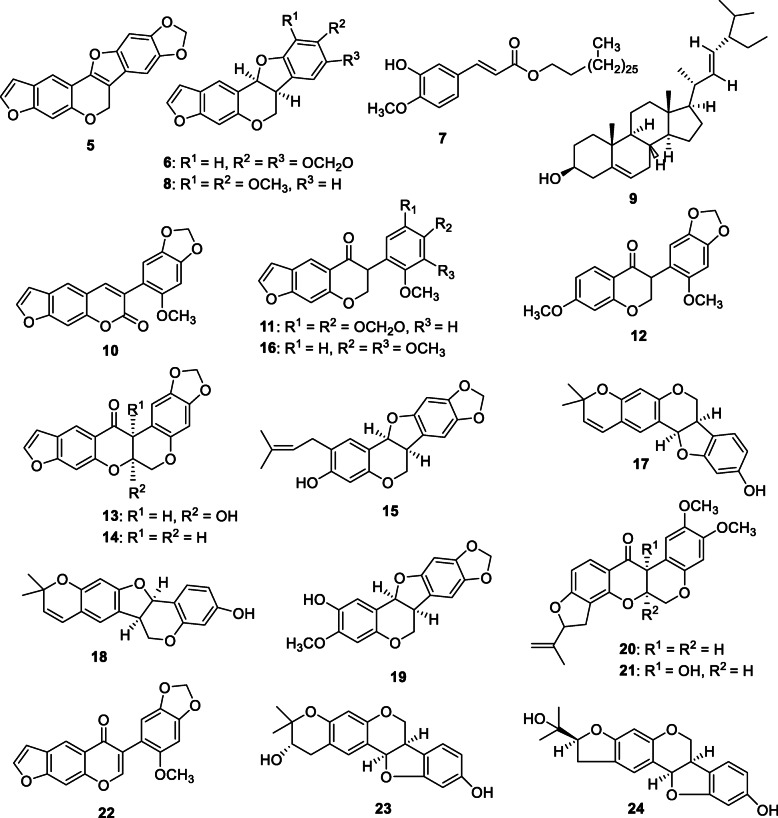
Compounds isolated from ABDCM (**5**–**24**)

The phytochemical studies on ABDCM lead to the isolation of compounds **5**–**24** (Fig. 2), namely; neoduleen (**5**), neodulin (**6**), ferulic acid (**7**), ambonane (**8**), stigmasterole (**9**), pachyrrhizine (**10**), neotenone (**11**), 7-methoxy-3-(6-methoxybenzo [d] [1,3] dioxol-5-yl) chroman-4-one (**12**), 12a-hydroxydolineon (**13**), dolineon (**14**), (−)-2-isopentenyl-3-hydroxy-8-9-methylenedioxypterocarpan (**15**), nepseudin (**16**), neorautenol (**17**), isoneorautenol (**18**), (−)-2-hydroxypterocarpin (**19**), rotenone (**20**), 12a-hydroxyrotenone (**21**), dehydroneotenone (**22**), rautandiol A (**23**) and rautandiol B (**24**), as described previously [11].

### Inhibitory activities of ABDCM and its compounds against *Plasmodium falciparum*, *Trypanosoma brucei rhodesiense*, and *Mycobacterium tuberculosis*

The ABDCM extract showed significant activity against two strains of *P. falciparum* giving IC_50_ values of 2.99 ± 0.59 μg/mL (SR - 0.9) and 2.67 ± 1.05 μg/mL (SR - 1.01) on TM4/8.2 and K1CB1, respectively. It also showed significant activity against *T. brucei rhodesiense* with an IC_50_ value of 3.04 ± 0.27 μg/mL. Compound **24** had significant inhibitory activities against both strains of *P. falciparum* with IC_50_ values of 0.40 ± 0.07 μM (SR - 108) and 0.74 ± 0.29 μM (SR - 133) against TM4/8.2 and K1CB1, respectively. Compounds **11**, **14**, **15**, **20**, and **21** also showed inhibitory activity against *T. brucei rhodesiense*, with the highest activity displayed by compound **15** having an IC_50_ value of 4.87 ± 0.49 μM (SR > 5.83). ABDCM and its compounds were not active against *M. tuberculosis* (Table 2).

**Table 2:** Inhibitory activities of crude extracts and isolated compounds against *P. falciparum, T. brucei rhodesiense*, and *M. tuberculosis*

Compounds (μM) Crude Extracts (μg/mL)	MTB H37Rv (MIC μM)	***T. br*** (IC_**50**_ μM)	***P. f*** (IC_**50**_ μM)	
			TM4/8.2	K1CB1
**ABDCM**	> 20	3.04 ± 0.27	2.99 ± 0.59 SR −0.9	2.67 ± 1.05 SR −1.01
**KEOH**	> 20	18.14 ± 1.34	> 50	> 50
**CEOH**	> 20	22.89 ± 1.29	> 50	> 50
**1**	> 20	8.74 ± 0.49 SR - 3.3	> 50	> 50
**2**	> 20	8.91 ± 0.03 SR - 4.52	> 50	> 50
**3**	> 20	> 100	> 100	> 100
**4**	> 20	> 100	> 100	> 100
**5**	> 62.1	–	> 31.0	> 31.0
**6**	> 64.9	–	> 32.5	> 32.5
**7**	> 34.4	–	> 17.2	> 17.2
**10**	> 64.7	–	> 32.3	> 32.3
**11**	> 62.0	17.01 ± 1.83	> 31.0	> 31.0
**13**	> 56.9	–	> 28.5	> 28.5
**14**	> 59.9	18.65 ± 3.20	> 29.6	> 29.6
**15**	> 56.8	4.87 ± 0.49 SR > 5.83	> 28.4	> 28.4
**16**	> 56.4	–	> 28.2	> 28.2
**19**	> 56.8	–	> 28.4	> 28.4
**20**	> 52.3	7.51 ± 0.17	> 26.2	> 26.2
**21**	> 55.2	8.63 ± 1.14	> 27.6	> 27.6
**22**	> 61.7	–	> 30.9	> 30.9
**24**	> 20	23.54 ± 1.59	0.40 ± 0.07 SR −108	0.74 ± 0.29 SR −133
**Isoniazid**	0.02	–	–	–
**Rifampin**	0.08	–	–	–
**Cycloguanil**	–	–	0.076 ± 0.016	10.7 ± 1.62
**Pyrimethamine**	–		0.095 ± 0.02	23.21 ± 2.24
**Pentamidine**	–	0.007 ± 0.00004	–	–

*Abbreviations*: *MTB Mycobacterium tuberculosis*, *T. br Trypanosoma brucei rhodesience*, *P. f Plasmodium falciparum*, *SR* Safety ratio

### Inhibitory activities of CEOH, KEOH and their compounds against *Plasmodium falciparum*, *Trypanosoma brucei rhodesiense*, and *Mycobacterium tuberculosis*

The CEOH and KEOH extracts showed moderate activities against *T. brucei rhodesiense* with IC_50_ values of 18.14 ± 1.34 μg/mL and 22.89 ± 1.29 μg/mL, respectively. Compounds **1** and **2**, which were isolated from CEOH, had significant inhibitory activities against *T. brucei rhodesiense* with IC_50_ values of 10.35 ± 0.38 μM (SR- 3.3) and 8.44 ± 0.16 μM (SR- 4.52), respectively. None of the compounds from KEOH were active against *T. brucei rhodesiense* and neither the extracts nor the compounds isolated from CEOH or KEOH were active against *M. tuberculosis* (Table 2).

### Inhibitory activity of the crude extracts and their compounds against α-glucosidase and α- amylase

**Fig. 3:**
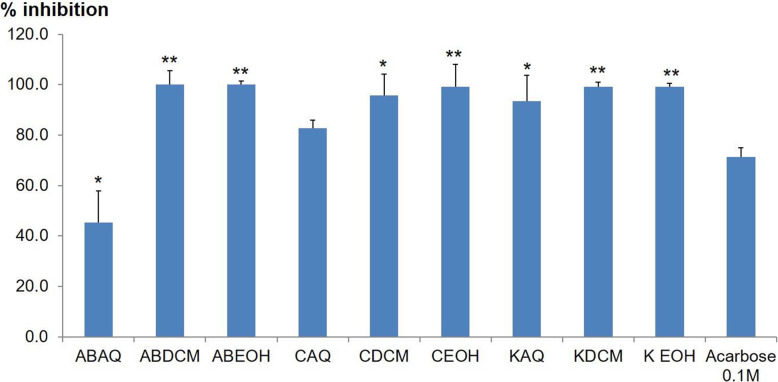
AGI activities of crude extracts at 500 μg/mL. Data are expressed as the mean ± SD. ANOVA analysis was performed in multiple comparisons to Acarbose 0.1 M. *p ≤ 0.05; **p ≤ 0.001

In this study, the crude extracts were screened for their AGI activities as shown in Fig. 3. The crude extracts from *N. mitis* (ABAQ, ABDCM and ABEOH), *S. surrattensis* (CAQ, CDCM and CEOH) and *H. abyssinica* (KAQ, KDCM and KEOH) show significantly higher percentage inhibitory activities than the positive control acarbose (71.3%) except the aqueous extract (ABAQ) from *N. mitis*, which gave a lower inhibitory activity of 45.4%. The highest inhibitory activity of 100% was from the DCM extract (ABDCM) from *N. mitis*.

**Fig. 4:**
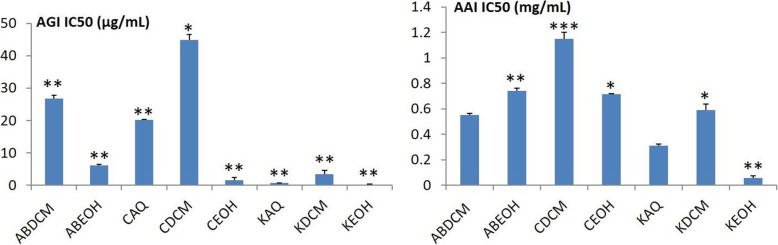
IC_50_ values of the most active crude extracts against AG and AA. Data are expressed as the mean ± SD. ANOVA analysis was performed in multiple comparisons to the IC_50_ of Acarbose against AG (438.5 ± 4.9 μg/mL) and AA (0.42 ± 0.07 mg/mL). *p ≤ 0.05; **p ≤ 0.001

The most active extracts were further analysed for their AAI and AGI activities. The data in Fig. 4 indicate that the ethanol crude extracts from *H. abyssinica* (KEOH) had the highest inhibitory activity with IC_50_ values of 0.21 ± 0.09 μg/mL against AG and 0.06 ± 0.02 mg/mL against AA. Both IC_50_ values were lower than that of acarbose which had IC_50_ values of 438.5 ± 4.9 μg/mL and 0.42 ± 0.07 mg/mL for AG and AA, respectively.

KAQ had an IC_50_ value of 0.7 ± 0.06 μg/mL and 0.31 ± 0.01 mg/mL against AG and AA, respectively. Also, the inhibitory activity of the DCM (ABDCM) and ethanol (ABEOH) extracts of *N. mitis* against AG gave significant IC_50_ values of 26.8 ± 1.01 μg/mL and 6.20 ± 0.32 μg/mL, respectively.

Of the compounds screened at 250 μg/mL for their AG inhibitory activities, compounds **3**, **14**, and **23** showed significant percentage inhibitory activities of 97.5 ± 0.6%, 54.2 ± 8.9% and 83.9 ± 1.0% when compared to the positive control acarbose that showed 86.5 ± 0.4% inhibition (Table 3).

**Table 3:** Screening of compounds for AGI activity

Samples (250 μg/mL) in 50% DMSO	% Inhibition
**3**	97.5 ± 0.6
**4**	47.7 ± 1.8
**7**	14.8 ± 0.8
**12**	17.7 ± 0.3
**14**	54.2 ± 8.9
**15**	31.5 ± 2.1
**21**	19.9 ± 11.4
**23**	83.9 ± 1.0
**24**	29.3 ± 3.1
**Acarbose 0.1 M**	86.5 ± 0.4
**DMSO 50%**	0

The IC_50_ values of these compounds were then determined against AG and AA (Table 4). Compound **3** (catechin) had modest IC_50_ values of 0.36 mM and 2.26 mM against AG and AA, respectively. Its inhibitory activity against AG was more than three times more potent than that of acarbose with an IC_50_ of 0.91 mM, however its potency against AA was three times less.

**Table 4:** IC_50_ values of the most active compounds against α-glucosidase (AG) and α-amylase (AA)

Compounds in 50% DMSO	AG IC_**50**_ (mM)	AA IC_**50**_ (mM)
**3**	0.36	2.26
**4**	1.38	ND
**14**	0.66	ND
**23**	0.28	ND
**Acarbose**	0.91	0.68

*ND* Not determined

Compounds **14** and **23** from ABDCM also showed moderate inhibitory activities against AG with IC_50_ values of 0.66 mM and 0.28 mM, respectively. Although the IC_50_ values of their inhibitory effect against AA were not determined, they may have played a role in the inhibitory activity displayed by the extract ABDCM.

## Discussion

The higher percentage yields obtained from the ethanol extracts can relate to the fact that both polar and semi-polar constituents of plant material can more readily dissolve in ethanol, leading to higher yields [55]. Previous phytochemical studies on *S. surrattensis*, collected from different locations, revealed the presence of quercetin, rutin, quercetin 3-*O*-glucoside 7-*O*- rhamnoside in the leaves [38], while 5,7-dihydroxy-4′-methoxyflavonol-3-*O*-β-D-galactopyranoside, chrysophanol, physcion, kaempferide, quercetin were isolated from extracts of the pods [56]. The bark yielded, chrysophanol and physcion, [57] while 8-hydroxy-6-methoxy-3-methylanthraquinone-1-*O*-α-L-rhamnopyranosyl (1–6)-β-D-glucopyranoside, chrysophanol, physcion, stearic acid, β-sitosterol and β-sitosterol-β-D-glucoside were isolated from the stem [58, 59]. The seeds yielded luteolin-7-*O*-β-D-glucopyranosyl-(1–4)-*O*-α-L-arabinopyranoside, γ-sitosterolin and digitolutein [60, 61]. Our studies shows that compounds **1** and **3** were isolated from this plant for the first time, the differences in the chemical constituents from the different parts of this plant may account for the variety of biological activities shown by this plant. Earlier reports of the phytochemical studies on the whole plant of *H. abyssinica*, found from different locations, revealed the presence of cirsiliol, *trans* 3,5-dihydroxy-4,7-dimethoxydihydroflavonol, vanillin, protocatechuic acid, catechin, stigmasterol, oleic acid, myristic acid and palmitric acid [17]. While, catechin, tyrosol and ethyl 3,4,-dihydroxybenzoate, were only isolated from the roots [25]. Our studies led to the isolation of salidroside for the first time from this plant.

Several other phytochemical studies on the roots of *N. mitis* have resulted in the isolation of neorautanone, 4-methoxyneoduline [14] 12a-hydroxyerosone, iseoliptol [15], neoraudiol [16], together with neodulin (**6**), pachyrrhizine (**10**), neotenone (**11**), 12a-hydroxydolineon (**13**), dolineon (**14**), rotenone (**20**), 12a-hydroxyrotenone (**21**), dehydroneotenone (**22**) [11, 14–16], nepseudin (**16**) [62], rautandiol A (**23**) and rautandiol B (**24**) [15]. Our earlier studies led to the isolation of one new compound (**12**) and nine known compounds including compounds **5**, **7**, **8**, **9**, **12**, **15**, **17**, **18** and **19**, which were isolated for the first time from *N. mitis* [11].

*P. falciparum*, *T. brucei rhodesiense*, and *M. tuberculosis*, are causative agents for malaria, trypanosomiasis and tuberculosis, respectively. These diseases are considered as re-emerging and their reoccurrence is either as a result of a breakdown in public health measures, the appearance of new strains of the causative organism or drug resistance [40, 41]. Due to the importance of this trend, it has become essential to invest in the alternative treatment to combat this re-emergence, and this can be achieved through the continuous search for new drug leads from natural sources. The screening of ABDCM showed significant inhibitory activity against *P. falciparum* and *T. brucei rhodesiense*, however, low safety ratios (SR) were observed in both strains of *P. falciparum* (TM4/8.2 and K1CB1) and this can be attributed to the earlier reports which suggested that the cytotoxicity of ABDCM may be caused by compounds **20** and **21**, as they were both cytotoxic [11, 15]. The significant inhibitory activity of compound **24** against the two strains of *P. falciparum* can account for the same activity showed by the ABDCM extract from which it was isolated. This demonstrates its potential as an antimalarial agent due to its activity against the mosquito vector and its larva [6, 12]. Importantly compound **24** was reported as non-cytotoxic on VERO and BHK21 cells [11]. With this safety range and excellent activity, compound **24** can be considered as a potential lead candidate for the development of a new anti-malaria drug.

ABDCM also showed significant inhibitory activity against *T. brucei rhodesiense*, compounds **11**, **14**, **15**, **20** and **21** are thought to be responsible for this activity, compound **15** gave the highest inhibitory activity and can serve as a lead candidate in the development of new therapy for trypanosomiasis. The inhibitory activity of ABDCM and its compounds against very important heamo-parasites like *P. falciparum* and *T. brucei rhodesiense* gives a lead for further investigations against other hemooparasites. Compounds **1** and **2** also gave moderate inhibitory activity against *T. brucei rhodesiense* and could be responsible for the same inhibitory activity shown by CEOH, from which these compounds were isolated. The inhibitory activities of the ABDCM and CEOH extracts against *T. brucei rhodesiense* are reported here for the first time based on our literature search.

All of the crude extracts from the selected plants except ABAQ, showed significant inhibitory activities against AG and AA, indicating their potential usefulness in the treatment of type II diabetes, which is a condition in which the capacity of the body to produce sufficient insulin is gradually lost. It is a degenerative condition in which the body becomes resistant to the normal effects of insulin, due mainly to excessive glucose absorption from the gastrointestinal tract (GIT) [63, 64]. The ability of the extracts to inhibit the digestive enzymes (AG and AA), which are responsible for the breakdown of starch or disaccharides and making glucose available for uptake by the small intestine, shows they are potential inhibitors of these enzymes which have been targeted in the effective management of glycaemia and maintaining glucose homeostasis [64–66].

The root crude extract of *H. abyssinica* was reported for its antioxidant and antiglycation activities, and these activities are also thought to be helpful in the management of diabetes complications [17]. The ethanol crude extract of *S. surrattensis* was previously screened for antidiabetic activity [37]. Both KEOH and CEOH showed significant inhibitory activity against AA and AG. Catechin (**3**) was isolated from both extracts and it is thought to be partly responsible for this activity. Catechin (**3**) is known for its bioactivities and health benefits, studies have revealed its ability to treat the symptoms of diabetes and its complications through the modification of oxidative stress [64, 65]. The observed modest inhibitory activities of the individual isolated phytochemicals indicate that other compounds or synergistic effects between the phytochemical components in these crude extracts may be responsible for the observed inhibitory activities against AG and AA.

## Conclusion

The extracts from the three selected medicinal plants have shown significant inhibitory activities against *P. falciparum, T. brucei rhodesiense*, α-amylase (AA) and α-glucosidase (AG). This study has revealed some of the individual compounds responsible for these inhibitory activities, giving indications for possible lead compounds for the development of new therapies against diseases caused by these agents. The inhibitory activity of the *N. mitis* compound **24** (rautandiol B) against *P. falciparum* was identified for the first time. It was found to be relatively safe based on its high safety ratio and therefore further studies on this compound are highly recommended for the development of alternative antimalarial therapy. Our study also revealed the importance of screening already identified and useful medicinal plants, as an aid in the discovery of new bioactivities which may be useful in the future development of new alternative therapeutic drugs.

## Supplementary Information

**Additional file 1.**

## Data Availability

The datasets used during the current study are available from the corresponding author on reasonable request.

## Abbreviations

MTB: *Mycobacterium tuberculosis*
Tbr: *Trypanosomea brucei rhodesiense*
MIC: Minimum concentration that causes 100% growth inhibition
IC_50_: Concentration that causes 50% growth inhibitory
HEPES: (4-(2-hydroxyethyl)-1-piperazineethanesulfonic acid)
DCM: Dichloromethane
CDCl_3_: Deuterated chloroform
AA: α-amylase
AG: α-glucosidase
CC: Column chromatography
PTLC: Preparative thin layer chromatography
TLC: Thin layer chromatography
