# Supplementary material for: Isolation of bioactive compounds from medicinal plants used in traditional medicine: Rautandiol B, a potential lead compound against Plasmodium falciparum
Source: BMC Complement Med Ther. 2021 Sep 13;21:231. doi: 10.1186/s12906-021-03406-y (PMC8438977; doi:10.1186/s12906-021-03406-y)
Supplement: Supplementary file 1 — Additional file 1. [file 12906_2021_3406_MOESM1_ESM.docx]

**Supporting Information**

**Title: Isolation of bioactive compounds from medicinal plants used in traditional medicine: Rautandiol B, a potential lead compound against *Plasmodium falciparum***

Christiana J. Dawurung^1,2^*, Minh T.H. Nguyen^3^, Jutharat Pengon^4^, Kanchana Dokladda^4^, Ratchanu Bunyong^4^, Roonglawan Rattanajak^4^, Sumalee Kamchonwongpaisan^4^, Phuong T.M. Nguyen^5^ and Stephen G. Pyne^1^.

^1^School of Chemistry and Molecular Bioscience, Faculty of Science Medicine and Health, University of Wollongong, Wollongong, New South Wales, 2522, Australia.

^2^ Department of Veterinary Physiology, Biochemistry and Pharmacology University of Jos, Jos Plateau State, Nigeria.

^3^Department of Life Science, University of Science and Technology of Hanoi, Vietnam Academy of Science and Technology, 18 Hoang Quoc Viet, Cau Giay, Hanoi, Vietnam.

^4^ National Center for Genetic Engineering and Biotechnology, National Science and Technology Development Agency, Pathum Thani, 12120, Thailand.

^5^Department of Plant Biochemistry, Institute of Biotechnology, Vietnam Academy of Science and Technology, 18 Hoang Quoc Viet, Cau Giay Hannoi, Vietnam.

**CONTENTS**

**Title Page**………………………………………………………………………………………....1

**^1^H NMR Spectra**………………………………………………….……………………………...5

**Figure 1** ^1^H NMR Spectrum of Compound **1** (400 MHz, CD_3_OD)…………………..........5

**Figure 2** ^1^H NMR Spectrum of Compound **2** (400 MHz, CD_3_OD)……………………......6

**Figure 3** ^1^H NMR Spectrum of Compound **3** (500 MHz, CD_3_OD)…………………….........7

**Figure 4** ^1^H NMR Spectrum of Compound **4** (400 MHz, CD_3_OD)………………………. 8

**Figure 5** ^1^H NMR spectrum of compound **5** (500 MHz, CDCl_3_)………………………...9

**Figure 6** ^1^H NMR spectrum of compound **6** (400 MHz, CDCl_3_)……………………….10

**Figure 7** ^1^H NMR spectrum of compound **7** (400 MHz, CDCl_3_)……………………….11

**Figure 8** ^1^H NMR spectrum of compound **8** (400 MHz, CDCl_3_)……………………….12

**Figure 9** ^1^H NMR spectrum of compound **10** (400 MHz, CDCl_3_)……………………...13

**Figure 10** ^1^H NMR spectrum of compound **11** (500 MHz, CDCl_3_)…………………….14

**Figure 11** ^1^H NMR spectrum of compound **12** (500 MHz, CDCl_3_)…………………….15

**Figure 12** ^1^H NMR spectrum of compound **13** ((400 MHz, CDCl_3_)……………………16

**Figure 13** ^1^H NMR spectrum of compound **14** (400 MHz, CDCl_3_)……………………17

**Figure 14** ^1^H NMR spectrum of compound **15** (500 MHz, CDCl_3_)…………………….18

**Figure 15** ^1^H NMR spectrum of compound **16** (500 MHz, CDCl_3_)…………………….19

**Figure 16** ^1^H NMR spectrum of compound **17** (400 MHz, CDCl_3_)…………………….20

**Figure 17** ^1^H NMR spectrum of compound **18** (500 MHz, CDCl_3_)………………….....21

**Figure 18** ^1^H NMR spectrum of compound **19** (400 MHz, CDCl_3_)…………………….22

**Figure 19** ^1^H NMR spectrum of compound **20** (500 MHz, CDCl_3_)…………………….23

**Figure 20** ^1^H NMR spectrum compound **21** (500 MHz, CDCl_3_)………………………..24

**Figure 21** ^1^H NMR spectrum compound **22** (400 MHz, CDCl_3_)………………………..25

**Figure 22** ^1^H NMR spectrum of compound **23** (500 MHz, MeOD)…………………….26

**Figure 23** ^1^H NMR spectrum of compound **24** (500 MHz, MeOD)…………………….27

**Physical and NMR Spectra Data for Compounds 1-24**………………………………………28

**Tables**……………………………………………………………………………………………28

**Table 1** Experimental and literature ^1^H NMR data of compound **1**……………………..28

**Table 2** Experimental and literature ^1^H NMR data of compound **2**……………………..29

**Table 3** Experimental and literature ^1^H NMR data of compound **3**……………………..30

**Table 4** Experimental and literature ^1^H NMR data of compound **4**……………………..31

**Table 5** Experimental and literature ^1^H NMR data of compound **5**……………………..32

**Table 6** Experimental and literature ^1^H NMR data of compounds **6**……………………33

**Table 7** Experimental and literature ^1^H NMR data of compound **7**……………………..34

**Table 8** Experimental and literature ^1^H NMR and ^13^C NMR data of compound **8**……...35

**Table 9** Experimental and literature ^1^H NMR and ^13^C NMR data of compound **9**……...36

**Table 10** Experimental and literature ^1^H NMR data of compound **10**…………………..37

**Table 11** Experimental and literature ^1^H NMR data of compound **11**…………………..38

**Table 12** ^1^H NMR and ^13^C NMR spectroscopic data of compound **12**………………….39

**Table 13** Experimental and literature ^1^H NMR data of compound **13**…………………..40

**Table 14** Experimental and literature ^1^H NMR data of compound **14**…………………..41

**Table 15** Experimental and literature ^1^H NMR data of compound **15**…………………..42

**Table 16** Experimental and literature ^1^H NMR data of compound **16**…………………..43

**Table 17** Experimental and literature ^1^H NMR data of compound **17**…………………..44

**Table 18** Experimental and literature ^1^H NMR data of compound **18**…………………..45

**Table 19** Experimental and literature ^1^H NMR data of compound **19**…………………..46

**Table 20** Experimental and literature ^1^H NMR data of compound **20**…………………..47

**Table 21** Experimental and literature ^1^H NMR data of compounds **21**…………………48

**Table 22** Experimental and literature ^1^H NMR data of compounds **22**…………………49

**Table 23** Experimental and literature ^1^H NMR data of compounds **23**…………………50

**Table 24** Experimental and literature ^1^H NMR data of compounds **24**…………………51

**Table 25** Experimental and literature ^13^C NMR data of

compounds **6, 10, 11, 13** and **14**………………………………………………………...52

**Table 26** Experimental and literature ^13^C NMR data of

compounds **18, 20, 21** and **22**……………………………………………………………53

**Table 27** Experimental and literature ^13^C NMR data of compounds **23** and **24**………...54

**References**……………………………………………………………………………………….55

**^1^H NMR Spectra of compounds 1-25**

**Figure 1** ^1^H NMR Spectrum of Compound **1** (400 MHz, CD_3_OD)

**Figure 2** ^1^H NMR Spectrum of Compound **2** (400 MHz, CD_3_OD)

**Figure 3**  ^1^H NMR Spectrum of Compound **3** (500 MHz, CD_3_OD)

**Figure 4** ^1^H NMR Spectrum of Compound **4** (400 MHz, CD_3_OD)

 **Figure 5** ^1^H NMR spectrum of compound **5** (500 MHz, CDCl_3_)

 **Figure 6** ^1^H NMR spectrum of compound **6** (400 MHz, CDCl_3_)

 **Figure 7** ^1^H NMR spectrum of compound **7** (400 MHz, CDCl_3_)

 **Figure 8** ^1^H NMR spectrum of compound **8** (400 MHz, CDCl_3_)

 **Figure 9** ^1^H NMR spectrum of compound **10** (400 MHz, CDCl_3_)

 **Figure 10** ^1^H NMR spectrum of compound **11** (500 MHz, CDCl_3_)

 **Figure 11** ^1^H NMR spectrum of compound **12** (500 MHz, CDCl_3_)

 **Figure 12** ^1^H NMR spectrum of compound **13** ((400 MHz, CDCl_3_)

 **Figure 13** ^1^H NMR spectrum of compound **14** (400 MHz, CDCl_3_)

 **Figure 14** ^1^H NMR spectrum of compound **15** (500 MHz, CDCl_3_)

 **Figure 15** ^1^H NMR spectrum of compound **16** (500 MHz, CDCl_3_)

 **Figure 16** ^1^H NMR spectrum of compound **17** (400 MHz, CDCl_3_)

 **Figure 17** ^1^H NMR spectrum of compound **18** (500 MHz, CDCl_3_)

 **Figure 18** ^1^H NMR spectrum of compound **19** (400 MHz, CDCl_3_)

 **Figure 19** ^1^H NMR spectrum of compound **20** (500 MHz, CDCl_3_)

 **Figure 20** ^1^H NMR spectrum compound **21** (500 MHz, CDCl_3_)

 **Figure 21** ^1^H NMR spectrum compound **22** (400 MHz, CDCl_3_)

 **Figure 22** ^1^H NMR spectrum of compound **23** (500 MHz, MeOD)

 **Figure 23** ^1^H NMR spectrum of compound **24** (500 MHz, MeOD)

**Physical and NMR Data for Compounds 1-25**

**Compound 1 (Kempherol)**^1^

Yellow powder; IR (neat) *v*_max_; 3221, 1607, 1517 1286 and 1020 cm^-1^; Mp 274-276 ^o^C, ^13^C NMR (101 MHz, MeOD) δ 175.9 (C-4), 164.3 (C-5), 161.1(C-7), 159.2 (C-4ʹ), 156.9 (C-8a), 146.7 (C-3), 135.7 (C-2), 129.3 (C-2ʹ 6ʹ), 122.3 (C-1ʹ), 114.91 (C-3ʹ 5ʹ), 103.1 (C-8b), 97.8 (C-6), 93.1 (C-8). For ^1^H NMR (400 MHz, CD3OD) spectroscopic data see Table 1.

**Table 1** Experimental and literature ^1^H NMR data of compound **1**

| **P** | **Kempherol ^1^H** δ **(CD3OD)** | |
| --- | --- | --- |
| 2ʹ 6ʹ  3ʹ 5ʹ  8  6 | **Expt. (400 MHz)**  8.10 (d, *J* = 9.0 Hz, 2H)  6.92 (d, *J* = 9.0 Hz, 2H)  6.41 (d, *J* = 2.1, 1H Hz)  6.20 (d, *J* = 2.1 Hz, 1H) | **Lit. (600 MHz, DMSO-*d*_6_)^1^**  8.04 (d, *J* = 9.0 Hz, 2H)  6.92 (d, *J* = 9.0 Hz, 2H)  6.44 (d, *J* = 1.8 Hz, 1H)  66.19 (d, *J* = 1.8 Hz, 1H) |

Assignments are made on the basis of COSY, HSQC, HMBC and NOESY correlations, Chemical shift values are in δ (ppm), and Coupling constants (*J*) are in Hz. P = Position

**Compound 2 (Quercetin)**^2^

Yellow solid obtained with some impurities; IR (neat) *v*_max_; 3234, 1606, 1517, 1285 and 1145 cm^-1^; Mp 292-296 ^o^C, (Lit-Mp 310-312 ^o^C),^187 13^C NMR (126 MHz, MeOD) δ 175.9 (C-4), 164.3 (C-7), 161.1 (C-5), 156.8 (C-8a), 147.4 (C-2), 146.5 (C-3'), 144.8 (C-4'), 135.8 (C-3), 122.7 (C-1'), 120.2 (C-6'), 114.8 (C-2'), 114.5 (C-5'), 103.0 (C-8b), 97.8, (C-8) 93.0 (C-6). For ^1^H NMR (400 MHz, CD3OD) spectroscopic data see Table 2.

**Table 2** Experimental and literature ^1^H NMR data of compound **2**

| **P** | **Quercetin ^1^H** δ **(CD3OD)** | |
| --- | --- | --- |
| 2ʹ  5ʹ  6  6ʹ  8 | **Expt. (400 MHz)**  7.75 (dd, *J* = 2.1, 1H)  6.91 (d, *J* = 8.6, 1H,)  6.41 (d, *J* = 2.1 Hz, 1H)  7.65 (dd, *J* = 8.5, 2.1, 1H)  6.20 (d, *J* = 2.0 Hz, 1H, H-8) | **Lit. (400 MHz, DMSO-*d*_6_)^2^**  7.64 (d, *J* = 8.5, 1H)  6.85 (d, *J* = 8.5, 1H)  6.37 (d,  *J* = 2.5, 1H)  7.49 (d,  *J* = 8.5, 1H)  6.14 (d,  *J* = 2.5, 1H) |

Assignments are made on the basis of COSY, HSQC, HMBC and NOESY correlations, Chemical shift values are in δ (ppm), and Coupling constants (*J*) are in Hz. P = Position

**Compound 3 (catachin)**^3,4^

Off white solid; IR (neat) *v*_max_; 3253, 1617, 1521, 1286 and 1029 cm^-1^; Mp 100.8-102.5 ^o^C, (Lit- 173-175 ^o^C)^4^ [α]_D_^24^ +15.4^°^ (*c* 0.4 MeOH), (Lit- [α]^24^_D_ +14.7^°^ (*c* 0.69 in acetone)^4^, MS (ESI +ve) *m/z* 289 ([M-H]^-^); HRMS (ESI +ve TOF) calcd for C_15_H_13_O_6_ 289.0719 found 289.0712 [M-H] ^-^; ^13^C NMR (126 MHz, MeOD) δ 156.5(C-7), 156.2 (C-5), 155.5 (C-8a), 144.9 (C-3′), 144.8 (C-4′), 130.8 (C-1′), 118.6 (C-6′), 114.7(C-2′), 113.9 (C-8b), 99.40 (C-6), 94.9(C-8), 81.46 (C-2), 67.42 (C-3), 27.13(C-4). For ^1^H NMR (500 MHz, CD_3_OD) spectroscopic data see Table **3**.

**Table 3** Experimental and literature ^1^H NMR data of compound **3**

| **P** | **Catechin ^1^H** δ **(CD_3_OD)** | |
| --- | --- | --- |
| 2  2ʹ  3  4  5ʹ  6  6ʹ  8 | **Expt. (500 MHz)**  4.61 – 4.53 (m, 1H)  6.86 (d, *J* = 2.0, 1H)  3.99 (td, *J* = 8.0, 5.5, 1H)  2.87 (dd, *J* = 16.1, 5.4, 1H)  2.52 (dd, *J* = 16.1, 8.2, 1H)  6.78 (d, *J* = 8.1, 1H)  5.87 (d, *J* = 2.3, 1H)  6.74 (dd, *J* = 8.3, 2.0, 1H)  5.95 (d, *J* = 2.3, 1H) | **Lit. (300 MHz)^3,4^**  4.55 (d, *J* = 7.5, 1H)  6.86 (d, *J* = 1.7, 1H)  3.98 (dt, *J* = 7.6, 5.3, 1H)  2.87 (dd, *J* = 5.5, 2H)  6.75 ( d, *J* = 8.1, 1H)  5.8 (d,  *J*= 2.2, 1H)  6.72 (d,  *J*=1.8, 1H)  5.9 ( d, *J* = 2.2, 1H) |

Assignments are made on the basis of COSY, HSQC, HMBC and NOESY correlations, Chemical shift values are in δ (ppm), and Coupling constants (*J*) are in Hz. P = Position

**Compound 4 (salidroside)**^5^

Brown solid; IR (neat) *v*_max_; 3221, 1607, 1520, 1285 and 1079 cm^-1^; Mp 128-130 ^o^C, (Lit-161-162 ^o^C)^5 13^C NMR (101 MHz, MeOD) δ 155.4 (C-3), 129.5 (C-6), 129.4 (C-1,2), 114.7 (C-4,5), 102.9 (C-4′), (C-8′) 76.5 (C-7′), 73.7 (C-5′), 70.7 (C-9′), 70.2 (C-6′), 61.4 (C-2′), 34.9 (C-1′). For ^1^H NMR (400 MHz, CD_3_OD) spectroscopic data see Table 4.

**Table 4** Experimental and literature ^1^H NMR data of compound **4**

| **P** | **salidroside ^1^H** δ **(CD3OD)** | |
| --- | --- | --- |
| 1,5  2, 4  1ʹ  2ʹ  4ʹ  5ʹ  6ʹ  7ʹ  8ʹ  9ʹ | **Expt. (400 MHz)**  7.08 (d, *J* = 8.5 Hz, 2H)  6.71 (d, *J* = 8.5 Hz, 2H)  2.89 – 2.82 (m, 2H)  3.85 (dd, *J* = 11.9, 1.9 Hz, 1H)  3.77 – 3.65 (m, 1H)  4.31 (d, *J* = 7.8 Hz, 1H)  3.20 (dd, *J* = 8.9, 7.9 Hz, 1H)  3.28 – 3.27 (m, 1H)  3.37 (m, 1H)  3.35 (m, 1H)  4.04 – 4.01 (m, 1H)  3.79 – 3.71 (m, 1H) | **Lit. (400 MHz, DMSO-*d*_6_)^5^**  7.04 (d, *J* = 8.3 Hz, 2H)  6.67 (d, *J* = 8.3 Hz, 2H)  2.73 (t, 8.6 Hz, 2H)  3.44 (dd, 11.4, 5.4Hz, 1H)  3.76 (d, 11.4Hz, 1H)  4.17 (d, *J* = 8.5 Hz, 1H)  3.15 (t, *J* = 8.5 Hz, 1H)  3.07 (m, 1H)  3.06 (d, *J* = 8.5, 1H)  2.96 (t,  *J* = 8.5, 1H)  3.87 (dd, *J* = 16.0, 8.6 Hz, 1H)  3.56 (dd, *J* = 16.0, 8.8 Hz, 1H) |

Assignments are made on the basis of COSY, HSQC, HMBC and NOESY correlations, Chemical shift values are in δ (ppm), and Coupling constants (*J*) are in Hz. P = Position

**Compound 5 (Neoduleen)**

White solid, ^13^C NMR (126 MHz, CDCl_3_) δ155.4 (C-3), 151.8 (C-4a), 150.6 (C-10a), 147.9 (C-11a), 145.9 (C-8), 144.8 (C-10), 144.7 (C-2ʹʹ), 121.6 (C-2), 119.0 (C-6a), 113.4 (C-1a), 111.9 (C-1), 108.3 (C-7a), 106.6 (C-3ʹʹ), 101.5 (C-2ʹʹʹ), 99.9 (C-4), 97.5 (C-7), 94.1 (C-10), 65.3 (C-6). For ^1^H NMR (500 MHz, CDCl_3_) spectroscopic data see Table 5.

**Table 5** Experimental and literature ^1^H NMR data of compound **5**

| **P** | **Neoduleen (5) ^1^H** δ **(CDCl_3_)** | |
| --- | --- | --- |
| 1  4  6  7  8  10  2ʹʹ  3ʹʹ  2ʹʹʹ | **Expt. (500 MHz)**  7.65 (s, 1H)  7.08 (s, 1H)  5.54 (s, 2H)  6.74 (s, IH)  --------  7.05 (s, 1H)  7.53 (d, *J* = 2.1, 1H)  6.73 (m, IH)  6.01 (s, 2H) | **Lit. (MHz)^6^**  7.63 (s)  7.06 (s)  5.51 (s, 2H)  6.75 (s)  --------  7.51 (s)  7.56 (d,  *J*= 2.2)  6.69 (d,  *J*=2.2)  5.98 (s, 2H) |

Assignments are made on the basis of COSY, HSQC, HMBC and NOESY correlations, Chemical shift values are in δ (ppm), and Coupling constants (*J*) are in Hz. P = Position

**Compound 6 (Neodulin)**^7^

White solid; IR (neat) *v*_max_; 1625 and 1453 cm^-1^; MS (ESI +ve) *m/z* 309 ([M+H]^+^); Mp 210^°^C; [α]^25^_D_ -176^°^ ( *c* 0.3 CHCl_3_); For ^1^H NMR (400 MHz, CDCl_3_) and ^13^C NMR (101 MHz, CDCl_3_) spectroscopic data see Tables 6 and 25 respectively.

**Table 6** Experimental and literature ^1^H NMR data of compounds **6**

| **P** | **Neodulin (6) ^1^H** δ **(CDCl_3_)** | |
| --- | --- | --- |
| 1  4  6  7  10  2ʹʹ  3ʹʹ  2ʹʹʹ  6a  11a | **Expt. (400 MHz)**  7.71 (s, 1H)  7.09 (s, 1H)  4.27 (ddd, *J*=11.0, 5.0, 0.5)  3.71 (t, *J* = 11.0, 1H)  6.74 (s)  6.44 (s, 1H)  7.55 (d, *J* = 2.2, 1H)  6.72 (dd, *J* = 2.2, 1.0, 1H)  5.92 ( d, *J* = 1.4, 1H)  5.89 (d, *J* = 1.4, 1H)  3.59 (dd, *J* = 7.2, 5.0, 1H)  5.66 (d, *J* = 7.2, 1H) | **Lit. (360 MHz)^7^**  7.72 (s)  7.09 (s)  4.28 (dd, *J* = 4.8, 10.8)  3.71 (dd, *J* = 10.8, 10.4)  6.75 (s)  6.44 (s)  7.56 (d,  *J* = 2.2)  6.72 (dd,  *J* = 2.2, 1.0)  5.90, 5.93 (d, *J* = 1.3)  --------  3.59 (ddd, *J* = 7.0, 4.8, 10.4)  5.67 (d,  *J* = 7.0) |

Assignments are made on the basis of COSY, HSQC, HMBC and NOESY correlations, Chemical shift values are in δ (ppm), and Coupling constants (*J*) are in Hz. P= Position

**Compound 7** (Ferulic Acid)

White solid, IR (neat) *v*_max_; 3536, 2848, 1700, 1517, 1265 and 734 cm^-1^; Mp 69-71^°^C; MS (ESI +ve) *m/z* 581 ([M+Na]^+^); HRMS (ESI +ve TOF) calcd for C_36_H_62_O_4_Na 581.4564 found 581.4563 [M+Na]^+^. For ^1^H NMR (400 MHz, CDCl_3_) and ^13^C NMR (101 MHz, CDCl_3_) spectroscopic data see Table 7.

**Table 7** Experimental and literature ^1^H NMR data of compound **7**

| **P** | **Ferulic acid Expt. (400 MHz, CDCl_3_)** | | **Lit. Ferulic acid (500 MHz)^8^** | |
| --- | --- | --- | --- | --- |
| 1  2  3  4  5  6  1ʹ  2ʹ  3ʹ  5ʹ  6ʹ  7ʹ  8ʹ  OCH_3_  OH | **^1^H NMR**  7.03 (d, *J* = 1.9, 1H)  6.91 (d, *J* = 8.1, 1H)  7.07 (dd, *J* = 8.2, 1.9, 1H)  7.61 (d, *J* = 15.9, 1H)  6.29 (d, *J* = 15.9, 1H)  4.19 (t, *J* = 6.7, 2H)  1.74 – 1.65 (m, 2H)  1.25 (s, 58H, H-7')  0.88 (t, *J* = 6.9 Hz, 3H)  3.93 (s, 3H)  5.86 (s, 1H) | **^13^C NMR**  127.1  114.7  146.8  147.9  109.3  123.1  144.6  115.7  167.4  64.6  29.7  29.7-22.7 (-CH_2_)_n_  14.1 (-CH_3_)  55.9  146.8 | **^1^H NMR**  7.04 (d, *J* = 1.8, 1H)  6.92 (d, *J* = 8.1, 1H)  7.08 (dd, *J* = 8.1, 1.81H)  7.62 (d, *J* = 15.9, 1H)  6.29 (d, *J* = 15.9, 1H)  4.20 (t, *J* = 6.7, 2H)  1.70 quint, J=6.8, 2H)  1.25 (s, 58H, H-7')  0.89 (t, *J* = 6.9, 3H)  3.94 (s, 3H)  5.85 (s. IH) | **^13^C NMR**  127.1  114.7  146.8  147.9  109.3  123.1  144.6  115.7  167.4  64.6  29.7  29.7-22.7 (-CH_2_)_n_  14.1 (-CH_3_)  55.9  ----- |

Assignments are made on the basis of COSY, HSQC, HMBC and NOESY correlations, Chemical shift values are in δ (ppm), and Coupling constants (*J*) are in Hz

**Compound 8 (Ambonane)**

White solid; IR (neat) *v*_max_; 2930, 1472 and 1268 cm^-1^; [α] ^25^_D_ -192^°^ (*c* 0.05 CHCl_3_) [Lit.^9^ [α]_D_ -214^°^ ( *c* 0.01 CHCl_3_)]; MS (ESI +ve) *m/z* 347 ([M+Na]^+^); HRMS (ESI +ve TOF) calcd for C_19_H_16_O_5_Na 347.0891 found 347.0895 [M+Na]^+^; ^13^C NMR (126 MHz, CDCl_3_) δ 155.7 (C-3), 153.5 (C-4a), 153.1 (C-9), 151.2 (C-10), 145.1 (2''), 145. (C-10a), 123.3 (C-7a), 122.3 (C-1), 121.7 (C-2), 118.1 (C-7), 116.4 (C-1a), 106.3 (C-3''), 104.8 (C-8), 99.7 (C-4), 79.7 (C-11a), 77.27, 77.01, 76.76, 66.9 (C-6), 60.7 (OCH_3_), 56.4 (OCH_3_), 40.1 (C-6a). For ^1^H NMR (400 MHz, CDCl_3_) spectroscopic data see Table **8**.

**Table 8** Experimental and literature ^1^H NMR and ^13^C NMR data of compound **8**

| **P** | **Ambonane(4) ^1^H** δ **(CDCl_3_)** | |
| --- | --- | --- |
| 1  4  6  7  8  2ʹʹ  3ʹʹ  6a  11a  OCH_3_ | **Expt. (400 MHz)**  7.81 (s, 1H)  7.09 (s, 1H)  4.33–4.27 (m)  3.73 (t, *J* = 10.7 1H)  6.91 (dd, *J* = 8.2,0.6 1H)  6.48 (d, *J* = 8.2, 1H)  7.56 (d, *J* = 2.2, 1H)  6.73 (dd, *J* = 2.2, 1.0 1H)  3.65 (dd, *J* = 6.2, 4.8 1H)  5.72 (d, *J* = 6.9 1H)  3.95 (s, 3H)  3.85 (s, 3H) | **Lit. (MHz)^9^**  7.71 (s)  7.03 (s)  4.22 (m)  3.72 (m)  6.84 (d)  6.41 (d)  7.50 (d,  *J* = 2.5)  6.63 (dd,  *J* = 2.5, 1.0)  3.53 (m)  5.69 (d)  3. 91 (s)  3.81 (s) |

Assignments are made on the basis of COSY, HSQC, HMBC and NOESY correlations, Chemical shift values are in δ (ppm), and Coupling constants (*J*) are in Hz

**Compound 9 (Stigmasterol)**^10^

White solid; IR (neat) *v*_max_; 3412, 2960, 1458, 1060 and 970 cm^-1^, MP 150-152^°^C; [α]^21^_D_ -43.2° (*c* 0.2 CHCl_3_); For ^1^H NMR (400 MHz, CDCl_3_) and ^13^C NMR (101 MHz, CDCl_3_) spectroscopic data see Table 9.

**Table 9** Experimental and literature ^1^H NMR and ^13^C NMR data of compound **9**

| **Position** | **Stigmasterol (5) ^1^H** δ **(CDCl_3_)** | | **Stigmasterol (5) ^1^H** δ **(CDCl_3_)** | |
| --- | --- | --- | --- | --- |
| 1  2  3  4  5  6  7  8  9  10  11  12  13  14  15  16  17  18  19  20  21  22  23  24  25  26  27  28  29 | **^1^H Expt. (400 MHz)**  3.51 (tdd, *J* = 4.5, 4.2, 3.8 1H)  5.31 (t, *J* = 6.1 1H)  0.91 (d, *J* = 6.2 3H)  4.98 (m, 1H)  5.14 (m, 1H)  0.83 (t, *J* = 7.1,3H)  0.82 (d, *J* = 6.6 3H)  0.80 (d, *J* = 6.6 3H)  0.71 (s, 3H)  1.03 (s, 3H) | **^13^C Expt.**  37.6  32.1  72.1  42.4  141.1  121.8  31.8  31.8  50.2  36.6  21.5  39.9  42.4  56.8  24.4  29.3  56.2  40.6  21.7  138.7  129.6  46.1  25.4  12.1  29.6  20.2  19.8  18.9  12.2 | **^1^H Lit. (600 MHz)^10^**  3.51 (tdd, *J* = 4.5, 4.2, 3.8 1H)  5.31 (t, *J* = 6.1 1H)  0.91 (d, *J* = 6.2 3H)  4.98 (m, 1H)  5.14 (m, 1H)  0.83 (t, 3H, *J* = 7.1 Hz)  0.82 (d, *J* = 6.6 3H)  0.80 (d, *J* = 6.6 3H)  0.71 (s, 3H)  1.03 (s, 3H) | **^13^C Lit.^10^**  37.6  32.1  72.1  42.4  141.1  121.8  31.8  31.8  50.2  36.6  21.5  39.9  42.4  56.8  24.4  29.3  56.2  40.6  21.7  138.7  129.6  46.1  25.4  12.1  29.6  20.2  19.8  18.9  12.2 |

Assignments are made on the basis of COSY, HSQC, HMBC and NOESY correlations, Chemical shift values are in δ (ppm), and Coupling constants (*J*) are inHz

**Compound 10 (Pachyrrhizine)**^7^

Yellow solid; IR (neat) *v*_max_; 2918, 1750, 1500 and 1269 cm^-1^; MS (ESI +ve) *m/z* 337 ([M+H]^+^); HRMS (ESI +ve TOF) calcd for C_19_H_13_O_6_ 337.0723 found 337.0712 [M+H]^+^; Mp 204-205^°^C; [α]^26^_D_ -189.5^°^ (*c* 0.01 CHCl_3_); For ^1^H NMR (400 MHz, CDCl_3_) and ^13^C NMR (101 MHz, CDCl_3_) spectroscopic data see Tables 10 and 25 respectively.

**Table 10** Experimental and literature ^1^H NMR data of compound **10**

| **P** | **Pachyrrhizine (10) ^1^H** δ **(CDCl_3_)** | |
| --- | --- | --- |
| 4  5  8  3ʹ  6ʹ  2ʹʹ  3ʹʹ  2ʹʹʹ  OCH_3_ | **Expt. (400 MHz)**  7.81 (s, 1H)  7.68 (s, 1H)  7.50 – 7.49 (m)  6.64 (s, 1H)  6.90 (s, 1H)  7.69 (d, *J*=2.3, 1H)  6.83 (dd, *J* = 2.3, 1.0,  1H)  5.97 (s, 2H)  3.78 (s, 3H) | **Lit. (360 MHz)^7^**  7.80 (s)  7.69 (s)  7.46 (brs)  6.64 (s)  6.91 (s)  7.70 (d,  *J* = 2.2)  6.76 (dd,  *J* = 2.2,  1.0)  5.98 (s)  3.79 (s) |

Assignments are made on the basis of COSY, HSQC, HMBC and NOESY correlations, Chemical shift values are in δ (ppm), and Coupling constants (*J*) are in Hz

**Compound 11 (Neotenone)**^7^

White needles; IR (neat) *v*_max_; 1682, 1474 and 1155 cm^-1^; Mp 174-175^°^C, [α]^26^_D_ -232.2^°^ ( *c*  0.4 CHCl_3_); For ^1^H NMR (400 MHz, CDCl_3_) and ^13^C NMR (101 MHz, CDCl_3_) spectroscopic data see Tables 11 and 25, respectively.

**Table 11** Experimental and literature ^1^H NMR data of compound **11**

| **P** | **Neotenone(11) ^1^H** δ **(CDCl_3_)** | |
| --- | --- | --- |
| 2  3  4  5  8  3ʹ  6ʹ  2ʹʹ  3ʹʹ  2ʹʹʹ  OCH_3_ | **Expt. (500 MHz)**  4.51 (dd, *J* = 10.9, 5.5 , 1H)  4.58 (t, *J* = 11.3, 1H)  4.32 (dd, *J* = 11.6, 5.5, 1H)  8.26 (s, 1H)  7.08 (s, 1H)  6.58 (s, 1H)  6.62 (s, 1H)  7.58(d, *J* = 2.0, 1H)  6.77 (brs, 1H)  5.92 (s, 2H)  3.73 (s, 3H) | **Lit. (360 MHz)^7^**  4.50 (dd, *J* = 5.4, 10.8)  4.58 (dd, *J* = 10.8, 11.4)  4.31 (dd, *J* = 5.4, 11.4 )  8.25 (s)  7.08 (brs)  6.57 (s)  6.62 (s)  7.57 (d,  *J* = 2.3)  6.76 (dd,  *J* = 2.3, 1.0)  5.90 (s)  3.72 (s) |

Assignments are made on the basis of COSY, HSQC, HMBC and NOESY correlations, Chemical shift values are in δ (ppm), and Coupling constants (*J*) are in Hz

**Compound 12 (7-methoxy-3-(6-methoxybenzo[*d*][1,3]dioxol-5-yl) chroman-4-one)**

Compound **12** was isolated and identified as a new compound. White solid, Mp 133-135^°^C, and [α]^22^_D_ -11.2 (*c* 0.2 CHCl_3_). Its HRESITOFMS data afforded an [M + H] + ion peak at *m/z* 329.1025, implying a molecular formula of C_18_H_16_O_6_ (calcd for C_18_H_17_O_6_, *m/z* 329.1025). For ^1^H NMR (500 MHz, CDCl_3_), ^13^C NMR (125 MHz, CDCl_3_) and HMBC spectroscopic data see Table 12.

**Table 12** ^1^H NMR and ^13^C NMR spectroscopic data of compound **12** (CDCl_3_) ^a^

| No | ^1^H NMR (500 MHz) | ^13^C NMR (125 MHz) | HMBC |
| --- | --- | --- | --- |
| 2  3  4  5  6  7  8  4a  8a  1'  2'  3'  4'  5'  6'  2''  OCH_3_2'  OCH_3_7 | 4.57 – 4.45 (m, 2H)  4.25 (dd, *J* = 11.3, 5.5, 1H)  7.92 (d, *J* = 8.8, 1H)  6.61 (dd, *J* = 8.8, 2.0, 1H)  6.44 (d, *J* = 2.0, 1H)  6.56 (s, 1H)  6.59 (s, 1H)  5.91 (s, 2H)  3.74 (s, 3H)  3.85 (s, 3H) | 71.3  47.5  191.5  129.4  109.9  165.8  100.7  115.5  163.8  115.6  152.8  95.3  141.3  147.7  109.7  101.3  56.6  56.6 | C-8a, 1', 3, 4  C-1', 2, 2', 4, 6'  C - 4, 7, 8a  C - 4a, 8  C- 6, 7, 8a  C - 4', 5'  C - 3, 2', 4', 2''  C - 4', 5'  C - 2'  C – 7 |
|  |  |  |  |

^a^Assignments were made on the basis of COSY, HSQC and HMBC correlations,

chemical shift values are in δ (ppm), and coupling constants (*J*) are in Hz.

**Compound 13 (12a-Hydroxydolineon)**^7^

Yellow solid; IR (neat) *v*_max_; 3460, 2918, 1624 and 1174 cm^-1^; MP 155-156^°^C, [α]^23^_D_ -173.2^°^ (*c* 0.3 CHCl_3_); For ^1^H NMR (400 MHz, CDCl_3_) and ^13^C NMR (101 MHz, CDCl_3_) spectroscopic data see Tables 7.9 and 25, respectively.

**Table 13** Experimental and literature ^1^H NMR data of compound **13**

| **P** | **12a-Hydroxydolineon (13) ^1^H** δ **(CDCl_3_)** | |
| --- | --- | --- |
| 1  4  6  8  11  2ʹʹ  3ʹʹ  2ʹʹʹ  6a  OH | **Expt. (400 MHZ)**  6.51 (s, 1H)  6.48 (s, 1H)  4.65 (dd, *J* = 11.8, 2.5,1H)  4.50 (d, *J* = 11.8, 1H)  7.02 (s, 1H)  8.19 (s, 1H)  7.55 (d, *J* = 2.0, 1H)  6.74 (d, *J* = 2.0, 1H)  5.85 (d, *J* = 1.0, 1H)  5.80 (d, *J* = 1.0, 1H)  4.62 (s, 1H)  4.42 (s, 1H) | **Lit. (360 MHz)^7^**  6.52 (s)  6.48 (s)  4.50 (dd, *J* = 12.9, 2.0 )  4.63 (dd, *J* = 12.0, 2.4)  7.02 (s)  8.19 (s, 1H)  7.54 (d, *J* = 2.2, 1H)  6.74 (dd, *J* = 2.2, 1.0, 1H)  5.86, 5.80 (d, *J* = 1.2)  4.62 (1Hbr)  4.46 (s) |

Assignments are made on the basis of COSY, HSQC, HMBC and NOESY correlations, Chemical shift values are in δ (ppm),

and Coupling constants (*J*) are in Hz. P= Position

**Compound 14 (Dolinion)**^7^

Brown solid; IR (neat) *v*_max_; 2924, 1625, 1467 and 1033 cm^-1^; MS (ESI +ve) *m/z* 359 ([M+Na]^+^); HRMS (ESI +ve TOF) calcd for C_19_H_12_O_6_Na 359.0529 found 359.0532 [M+Na]^+^; Mp 220^°^C; [α]^23^_D_ +139.7^°^ ( *c* 0.2 CHCl_3_); For ^1^H NMR (400 MHz, CDCl_3_) and ^13^C NMR (101 MHz, CDCl_3_) spectroscopic data see on Tables 14 and 25, respectively.

**Table 14** Experimental and literature ^1^H NMR data of compound **14**

| **P** | **Dolineon (10) ^1^H** δ **(CDCl_3_)** | |
| --- | --- | --- |
| 1  4  6  8  11  2ʹʹ  3ʹʹ  2ʹʹʹ  6a  12a | **Expt. (400 MHz)**  6.73 (s, 1H)  6.44 (s, 1H)  4.63 (dd, *J* = 12.0, 3.2, 1H)  4.19 (d, *J* = 12.0, 1H)  7.06 (s, 1H)  8.21 (s, 1H)  7.54 (d, *J* = 2.3, 1H)  6.74 (dd, *J* = 2.3, 1.0, 1H)  5.86 (d, *J* = 1.4, 1H),  5.80 (d, *J* = 1.4, 1H)  4.99 – 4.92 (m, 1H)  3.89 (d, *J* = 3.9, 1H) | **Lit. (360 MHz)^7^**  6.72 (s) overlap  6.44 (s)  4.63 (d, *J* = 12.0, 3.2)  4.19 (dd, *J* = 12.0)  7.05 (br s)  8.21 (s)  7.54 (d,  *J* = 2.3)  6.73 (dd,  *J* = 2.3, 1.0)  5.80, 5.86 (d, *J* = 1.3)  4.96 (ddd, *J* = 3.2, 1.0, 3.9)  3.89 (d,  *J* = 3.9) |

Assignments are made on the basis of COSY, HSQC, HMBC and NOESY correlations, Chemical shift values are in δ (ppm),

and Coupling constants (*J*) are in Hz. P= Position

**Compound 15 (-)-2-isopentenyl-3-hydroxy-8-9-methylenedioxypterocarpan**^11^

White sticky solid; IR (neat) *v*_max_; 3364, 2921, 1603, 1476, 1116 and 834 cm^-1^; Mp 149-152^°^C (Lit.^11^ 146-147^°^C), [α]^23^ -339.5^°^ (*c* 0.1 CHCl_3_) [Lit.^11^ [α]^23^_D_ -261.9^°^ (*c* 0.8 CHCl_3_), ^13^C NMR (126 MHz, CDCl_3_) δ 155.8 (C-3), 154.9 (C-4a), 154.2 (C-10a), 148.1 (C-9a), 141.7 (C-8a), 134.8 (C-3'), 131.8 (C-1), 121.9 (C-2'), 121.2 (C-3), 118.1 (C-7a), 112.2 (C-1a), 104.8 (C-7), 103.9 (C-4), 101.3 (C-2''), 93.8 (C-10), 78.6 (C-11a), 77.28, 77.03, 76.77, 66.5 (C-6), 40.2 (C-6a), 29.2 (1'), 25.8 (C-5'), 17.9 (C-4'). For ^1^H NMR (500 MHz, CDCl_3_) spectroscopic data see Table 15.

**Table 15** Experimental and literature ^1^H NMR data of compound **15**

| **P** | **(-)-2-isopentenyl-3-hydroxy-8-9-methylenedioxypterocarpan (15) ^1^H** δ **(CDCl_3_)** | |
| --- | --- | --- |
| 1  4  6  7  10  1ʹ  2ʹ  4ʹ  5ʹ  2ʹʹ  6a  11a  OH | **Expt. (500 MHz)**  7.21 (s, 1H)  6.44 (s, 1H)  4.19 (dd, = 11.0, 5.0, 1H)  3.61 (t, *J* = 11.0, 1H)  6.71 (s, 1H)  6.39 (s, 1H)  3.32 (d, *J* = 7.2, 2H)  5.34 – 5.29 (m, 1H)  1.81 (S, 3H)  1.76 (S,3H)  5.88, 5.91, (d, *J* = 1.4, 2H)  3.44 (s, 1H)  5.45 (d, *J* = 6.9, 1H)  5.37 (d, *J* = 4.4, 1H) | **Lit.^11^**  7.25 (s)  6.47 (s)  4.18  3.62  6.73 (s)  6.42 (s)  3.27-3.38 (d)  5.45-5.22 (m)  1.77 (s)  1.71 (s)  5.92  3.47 (s)  5.46  5.45 |

Assignments are made on the basis of COSY, HSQC, HMBC and NOESY correlations, Chemical shift values are in δ (ppm), and Coupling constants (*J*) are in Hz. P= Position

**Compound 16 (Nepseudin)**^12^

White sticky solid; IR (neat) *v*_max_; 2919, 1687, 1465 and 1260 cm^-1^ [α]^24^_D_ -102.2^°^ (*c* 0.8 CHCl_3_), [Lit.^12^ [α]^20^ 0^°^ (CHCL_3_)], MS (ESI +ve) *m/z* 377 ([M+Na]^+^); HRMS (ESI +ve TOF) calcd for C_20_H_18_O_6_Na 377.1001 found 377.0992 [M+Na]^+^ ; ^13^C NMR (101 MHz, CDCl_3_) δ 192.9 (C-4), 159.9 (C-8a), 159.3 (C-7), 153.7 (C-3'), 152.0 (C-2'), 146.0 (C2''), 142.3 (C-4'), 124.5 (C-5'), 122.7 (C-6), 121.5 (C-1'), 120.9 (C-5), 118.8 (C-4a), 107.4 (C-6'), 107.1 (C-3''), 99.7 (C-8), 71.6 (C-2), 60.7 (OCH_3_), 60.7 (OCH_3_), 56.0 (OCH_3_), 49.0 (C-3). For ^1^H NMR (400 MHz, CDCl_3_) spectroscopic data see Table 16.

**Table 16** Experimental and literature ^1^H NMR data of compound **16**

| **P** | **Nepseudin (16) ^1^H** δ **(CDCl_3_)** | |
| --- | --- | --- |
| 2  3  5  8  5ʹ  6ʹ  2ʹʹ  3ʹʹ  OCH_3_ | **Expt. (400MH)**  4.64 (dd, *J*= 11.9, 11, 1H)  4.52 (dd, *J*= 11.0, 5.5, 1H)  4.24 (dd, *J*=11.9,5.5, 1H)  8.27 (s, 1H)  7.06 (s, 1H)  6.84 (d, *J* = 8.5, 1H)  6.65 (d, *J* = 8.5, 1H)  7.59 (d, *J*=2.3, 1H)  6.77 (dd, *J* = 2.3, 1.0 1H)  3.87 (s, 3H)  3.86 (s, 3H)  3.85 (s, 3H) | **Lit.^12^**  4.50 (dd)  4.50 (dd)  4.50 (dd)  8.23 (s)  7.07 (s)  6.83 (d)  6.75 (d)  7.56 (d)  6.70 (d)  3.90 (s)  3.88 (s)  3.87 (s) |

Assignments are made on the basis of COSY, HSQC, HMBC and NOESY correlations, Chemical shift values are in δ (ppm), and Coupling constants (*J*) are in Hz

**Compound 17 (Neorautenol)**^6,13^

White sticky solid; IR (neat) *v*_max_; 3397, 2925, 1622, 1492, 1134 and 958 cm^-1^; Mp 172-174 ^°^C, (Lit.^6,13^ 93-95 ^°^C) [α] ^21^_D_ -127.2^°^ (*c* 0.2 CHCl_3_) [Lit. ^6,13^ [α]_D_ -273^°^ (*c* 0.08 in CHCl_3_)]_,_ MS (ESI +ve) *m/z* 345 ([M+Na]^+^); HRMS (ESI +ve TOF) calcd for C_20_H_18_O_4_Na 345.1109 found 345.1103 [M+Na]^+^ ; ^13^C NMR (101 MHz, MeOD) δ 161.9 (C-10a), 159.8 (C-9), 157.8 (C-3), 155.7 (C-4a), 130.2 (C-3'), 129.8 (C-1), 125.9 (C-7), 122.7 (C-4'), 119.4 (C-7a), 117.5 (C-1a), 114.2 (C-2), 108.7 (C-8), 105.3 (C-4), 98.8 (C-10), 79.7 (C-2'), 77.6 (C-11a), 67.7 (C-6), 40.9 (C-6a), 28.4 (3''), 28.2 (2''). For ^1^H NMR (400 MHz, CD_3_OD) spectroscopic data see Table 17.

**Table 17** Experimental and literature ^1^H NMR data of compound **17**

| **P** | **Neorautenol (17) ^1^H** δ **(CD_3_OD)** | |
| --- | --- | --- |
| 1  4  6  7  8  10  11a  3ʹ  4ʹ  5ʹ  2ʹʹ  4ʹʹ  5ʹʹʹ | **Expt. (400MHz)**  7.11 (s, 1H)  6.26 (s, 1H)  4.22 (dd, *J* = 9.7, 3.6, 1H)  3.51 (dd, *J* = 7.2, 5.7, 1H)  7.07 (d, *J* = 8.0, 1H)  6.33 (dd, *J* = 8.0, 2.1, 1H)  6.29 (d, *J* = 2.1, 1H)  5.42 (d, *J* =6.3, 1H)  5.61 (d, *J* = 9.8, 1H)  6.37 (d, *J* = 9.8, 1H)  1.39 (s, 3H)  1.40 (s, 3H)  3.36 – 3.27 (m, 1H)  2.78 (s) | **Lit.^6,13^**  7.16 (s)  6.42 (s)  4.22 (m)  3.62 (m)  7.06 (d)  6.36 (d)  6.40 (d)  5.49 (d)  5.56 (d)  6.33 (d)  1.41 (s, 3H)  1.43 (s, 3H)  3.52 (m) |

Assignments are made on the basis of COSY, HSQC, HMBC and NOESY correlations, Chemical shift values are in δ (ppm), and Coupling constants (*J*) are in Hz. P= Position

**Compound 18 (Isoneorautenol)**^14^

Sticky white; IR (neat) *v*_max_; 3397, 2916, 1706, 1623, 1264 and 1156 cm^-1^; MS (ESI +ve) *m/z* 323 ([M+H]^+^); HRMS (ESI +ve TOF) calcd for C_20_H_19_O_4_ 323.1283 found 323.1284 [M+H]^+^ ; [α]^21^_D_ -234.6^°^ (*c* 0.1 CHCl_3_); For ^1^H NMR (500 MHz, CDCl_3_) and ^13^C NMR (126 MHz, CDCl_3_) spectroscopic data see on Tables 18 and 26, respectively.

**Table 18** Experimental and literature ^1^H NMR data of compound **18**

| **P** | **Isoneorautenol (18) ^1^H** δ **(CD_3_OD)** | |
| --- | --- | --- |
| 2  3  4  5  6  8  2ʹ  5ʹ  3ʹʹ  4ʹʹ  4ʹʹʹ  5ʹʹʹ  OH | **Expt. (400 MHz)**  4.23 (dd, *J* = 11.0, 5.1, 1H)  3.62 (t, *J* = 11.0, 1H,)  3.49 (dd, *J* = 11.0, 5.1, 1H)  5.49 – 5.42 (m, 1H)  7.39 (d, *J* = 8.3, 1H,)  6.55 (dd, *J* = 8.3, 2.2 1H)  6.41 (d, *J* = 2.2, 1H)  6.84 (s, 1H)  6.33 (s, 1H)  6.36 (d, *J* = 9.8, 1H)  6.25 (d, *J* = 9.8, 1H)  1.57 (s, 3H)  1.41 (s, 3H)  2.75 (s) | **Lit. (300 MHz)^14^**  4.21 (t, *J* = 10.8, 1H)  3.41 (dd *J* = 10.8, 4.8, 1H)  3.49 (m, 1H)  5.45 (d, *J* = 10, 1H)  7.37 (d, J = 8.4, 1H)  6.53 (dd, *J* = 8.4, 1.5, 1H)  6.40 (d, *J* = 1.5, 1H)  6.84 (s, 1H)  6.33 (s. 1H)  6.37 (d, *J* = 6.8, 1H)  6.24 (d, *J* = 10, 1H)  1.42 (s, 3H)  1.40 (s, 3H)  ------- |

Assignments are made on the basis of COSY, HSQC, HMBC and NOESY correlations, Chemical shift values are in δ (ppm), and Coupling constants (*J*) are in Hz. P= Position

**Compound 19 ((-)-2-Hydroxypterocarpin)**^11^

White powder; IR (neat) *v*_max_; 3495, 2919, 1512, 1456 and 1216 cm^-1^; Mp 223-225 ^°^C (Lit.^11^ 238-239 ^°^C) [α]^23^ -107.4^°^(*c* 0.3 CHCl_3_) [Lit.^11^ [α] ^23^_D_ -227.7^°^ (*c* 0.8 CHCl_3_)]; MS (ESI +ve) *m/z* 315 ([M+H]^+^); HRMS (ESI +ve TOF) calcd for C_17_H_15_O_6_ 315.0882 found 315.0883 [M+H]^+^ ; ^13^C NMR (101 MHz, Acetone) δ 154.4 (C-10a), 149.1 (C-4a), 147.9 (C-9), 141.6 (C-8), 143.3 (C-3), 141.3 (C-2), 118.6 (C-7a), 115.7 (C-1), 112.1 (C-1a), 104.9 (C-7), 101.2 (C-2''), 100.4 (C-4), 93.1 (C-10), 78.5 (C-11a), 66.3 (C-6), 55.3 (OCH_3_). For ^1^H NMR (400 MHz, Acetone *d*_6_) spectroscopic data see Table 19.

**Table 19** Experimental and literature ^1^H NMR data of compound **19**

| **P** | **(-)-2-hydroxypterocarpin (19) ^1^H** δ **(Acetone *d*_6_)** | |
| --- | --- | --- |
| 1  4  6  7  10  2ʹʹ  6a  11a  OCH_3_  OH | **Expt. (400 MHz)**  6.89 (s, 1H)  6.40 (s, 1H )  4.27 – 4.23 (m)  3.65 – 3.58 (m)  6.90 (s, 1H)  6.47 (s, 1H)  5.92 (d, *J* = 1.0, 2H)  3.71 – 3.47 (m, 1H)  5.47 (d, *J* = 6.9, 1H)  3.82 (s, 3H)  5.62 (s) | **Lit.^11^**  7.01 (s)  6.44 (s)  4.22 (m)  3.65 (m)  6.70 (s)  6.42 (s)  5.90 (d, 2H)  3. 45 (m)  5.43  3.87 (s)  5.28 (s) |

Assignments are made on the basis of COSY, HSQC, HMBC and NOESY correlations, Chemical shift values are in δ (ppm), and Coupling constants (*J*) are in Hz. P= Position

**Compound 20 (Rotenone)**^15,16^

Yellow needles; IR (neat) *v*_max_; 2920, 1606, 1455, 1196 and 815 cm^-1^; MS (ESI +ve) *m/z* 395 ([M+H]^+^); HRMS (ESI +ve TOF) calcd for C_23_H_23_O_6_ 395.1495 found 395.1490 [M+H]^+^ ; Mp 159-162^°^C; [α]^24^_D_ -236^°^ (*c* 0.3 CHCl_3_); ^1^H NMR (500 MHz, CDCl_3_) and ^13^C NMR (126 MHz, CDCl_3_) spectroscopic data see on Tables 20 and 26, respectively.

**Table 20** Experimental and literature ^1^H NMR data of compound **20**

| **P** | **Rotenone (20) ^1^H** δ **(CDCl_3_)** | |
| --- | --- | --- |
| 1  4  6  10  11  4ʹ  5ʹ  7ʹ  8ʹ  6a  12a  OCH_3_ | **Expt. 400MHz**  6.77 (s, 1H)  6.45 (s, 1H)  4.67 – 4.55 (m, 1H)  4.18 (d, *J* =12.0 Hz, 1H)  6.51 (d,*J*=8.6, 1H)  7.84 (d, *J* = 8.5, 1H)  3.32 (dd,*J*= 15.7, 9.8 1H)  2.96 (dd, *J* =15.7, 1H)  5.24 (t, *J* = 8.9 1H)  5.07 (s, 1H)  4.94 (s, 1H)  1.77 (s, 3H)  4.93 (d, *J* = 3.7, 1H)  3.84 (s, 1H)  3.81 (s)  3.77 (s) | **Lit. (360 MHz)^15,16^**  6.76 (s)  6.43 (s)  4.59 (m)  4.15 (m)  6.48 ( d)  7.82 (d)  3.33 (m)  2.94 (m)  5.24 (m)  5.05 (s)  4.94 (s)  1.77 (s)  4.96 (m)  3.79 (s)  3.88 (s)  3.74 (s) |

Assignments are made on the basis of COSY, HSQC, HMBC and NOESY correlations, Chemical shift values are in δ (ppm), and Coupling constants (*J*) are in Hz.P= Position

**Compound 21 (12a- Hydroxyrotenone)^7,15^**

Brown solid; IR (neat) *v*_max_; 3468, 2923, 1604, 1455, 1203, 1088 and 814 cm^-1^; MP 152-154^°^C; [α]^20^_D_ -59.2^°^ (*c* 0.5 CHCl_3_); For ^1^H NMR (500 MHz, CDCl_3_) and ^13^C NMR (126 MHz, CDCl_3_) spectroscopic data see on Tables 21 and 26, respectively.

**Table 21** Experimental and literature ^1^H NMR data of compounds **21**

| **P** | **12a- hydroxyrotenone (21) ^1^H** δ **(CDCl_3_)** | |
| --- | --- | --- |
| 1  4  6  10  11  4ʹ  5ʹ  7ʹ  8ʹ  6a  OCH_3_  OH | **Expt. (500 MHz)**  6.55 (s, 1H)  6.48 (s, 1H)  4.61 (d, *J* = 2.5,1H)  4.60 – 4.57 (m, 1H)  6.54 (d,*J*=8.6, 1H)  7.83 (d, *J* = 8.6, 1H)  2.94 (dd,*J*= 15.8, 8.2,1H) 3.29(dd, *J* = 15.8, 9.8, 1H)  5.24 (t, *J* = 9.0,1H)  5.06 (s, 1H)  4.94 (s, 1H)  1.76 (s, 3H)  4.47 (d, *J* = 4.8, 1H)  3.82 (s)  3.72 (s)  4.50 (s) | **Lit. (360 MHz)^7,15^**  6.55 (s)  6.48 (s)  4.50 (m)  4.62 (m)  6.53 (d, *J*=8.5)  7.70 (d, *J*=8.5)  2.94 (m)  3.29 (m)  5.20 (m)  5.07 (s)  4.94 (s)  1.76 (s, br)  4.58 (m)  3.78 (s)  3.70 (s)  4.5 (br) |

Assignments are made on the basis of COSY, HSQC, HMBC and NOESY correlations, Chemical shift values are in δ (ppm), and Coupling constants (*J*) are in Hz.P= Position

**Compound 22 (Dehydroneotenone)**^7^

White fluffy; IR (neat) *v*_max_; 2933, 1623, 1472 and 1194 cm^-1^; MP 237-239^°^C; [α]^26^_D_ -152.6^°^ (*c* 0.1 CHCl_3_); For ^1^H NMR (400 MHz, CDCl_3_) and ^13^C NMR (101 MHz, CDCl_3_) spectroscopic data see on Tables 22 and 26, respectively.

**Table 22** Experimental and literature ^1^H NMR data of compounds **22**

| **P** | **Dehydroneotenone (22) ^1^H** δ **(CDCl_3_)** | |
| --- | --- | --- |
| 2  5  8  3ʹ  6ʹ  2ʹʹ  3ʹʹ  2ʹʹʹ  OCH_3_ | **Expt. (400 MHz)**  7.99 (s, 1H)  8.54 (s, 1H)  7.58 (s, 1H)  6.64 (s, 1H)  6.86 (s, 1H)  7.73 (d, *J* = 2.3, 1H)  6.91 (dd, *J* = 2.3, 1.0, 1H)  5.96 (s, 2H)  3.74 (s, 3H) | **Lit. (360 MHz)^7^**  7.98 (s)  8.28 (s)  7.63 (brs)  6.64 (s)  6.70 (s)  7.88 (d,*J*=2.0)  6.93 (dd, *J* = 2.0, 1.0)  5.86 (s)  3.78 (s) |

Assignments are made on the basis of COSY, HSQC, HMBC and NOESY correlations, Chemical shift values are in δ (ppm), and Coupling constants (*J*) are in Hz

**Compound 23 (Rautandiol A)**^17^

White solid; IR (neat) *v*_max_; 3404, 2923, 1626, 1486, 1123 and 958 cm^-1^; MS (ESI +ve) *m/z* 363 ([M+Na]^+^); HRMS (ESI +ve TOF) calcd for C_20_H_20_O_5_Na 363.1208 found 363.1218 [M+Na]^+^ ; Mp 194-196^°^C, [α]^21^_D_ -161.4^°^ (*c* 0.1 MeOH); For ^1^H NMR (500 MHz, CD_3_OD) and ^13^C NMR (126 MHz, CD_3_OD) spectroscopic data see on Tables 23 and 27, respectively.

**Table 23** Experimental and literature ^1^H NMR data of compounds **23**

| **P** | **Rautandiol A (23)^1^H** δ **(CD_3_OD)** | |
| --- | --- | --- |
| 1  4  6  7  8  10  6a  11a  1'  2'  5'  4'  OH | **Expt. (500 MHz)**  7.17 (s)  6.26 (s)  4.21 – 4.15 (m)  3.57 – 3.44 (m)  7.06 (d, 8.1)  6.32 (dd, *J* = 8.0, 2.2)  6.24 (d, *J* = 2.2)  3.48 (m)  5.43 (d, *J* = 6.5)  3.00 ( dd, *J* =16.6, 5.3)  2.70 (ddd, *J* = 16.3, 7.7, 0.7)  3.75 (dd, 7.7, 5.3)  1.33 (s)  1.24 (s)  4.59 (s) | **Lit.^17^**  7.17 (s)  6.26 (s)  4.18 (dd, *J* = 9.6, 3.1)  3.51-3.46 (m)  7.06 (d, *J* = 8.1)  6.31 (dd, *J* = 8.1, 2.4)  6.23 (d, *J* = 2.4)  3.50-3.46 (m)  5.44 (d, *J* = 6.6)  3.00 (dd, *J* = 16.5, 5.3)  2.69 (dd, *J* = 16.5, 7.8)  3.74 (dd, *J* = 7.8, 5.3)  1.33 (s)  1.24 (s)  ------- |

Assignments are made on the basis of COSY, HSQC, HMBC and NOESY correlations, Chemical shift values are in δ (ppm), and Coupling constants (*J*) are in Hz

**Compound 24 (Rautandiol B)**^17^

White solid; IR (neat) *v*_max_; 3517, 3202, 1617, 1484, 1153 and 833 cm^-1^; MS (ESI +ve) *m/z* 341 ([M+H]^+^); HRMS (ESI +ve TOF) calcd for C_20_H_21_O_5_ 341.1489 found 341.1304 [M+H]^+^ ; Mp 209- 213^°^C; [α]^21^_D_ -208.5^°^ (*c* 0.1 MeOH); For ^1^H NMR (500 MHz, CD_3_OD) and ^13^C NMR (126 MHz, CD_3_OD) spectroscopic data see on Tables 20 and 27, respectively.

| **P** | **Rautandiol B (24) ^1^H** δ **(CD_3_OD)** | |
| --- | --- | --- |
| 1  4  6  7  8  10  6a  11a  1'  4'  6'  OH | **Expt. (500 MHz)**  7.25 (s)  6.26 (s)  4.20 ( dd, *J* = 10.0,3.8)  3.54 – 3.45 (m)  7.07 (d, *J* = 8.1)  6.32 (dd, *J* = 8.0, 2.2)  6.23 (s)  3.48 (m)  5.45 (d, *J* = 6.5)  3.14 (d, *J* = 8.7)  1.22 (s)  1.24 (s)  -------- | **Lit.^17^**  7.24 (s)  6.26 (s)  4.19 (dd, *J* = 10.0, 3.6)  3.50 (dd, *J* = 10.0, 10.0)  7.06 (d, *J* = 8.1)  6.31 (dd, *J* = 8.1, 2.2)  6.23 (d, *J* = 2.2)  3.48-3.44 (m)  5.43 (d, *J* = 6.6)  3.13 (d, *J* = 8.9)  1.21 (s)  1.24 (s)  -------- |

**Table 24** Experimental and literature ^1^H NMR data of compounds **24**

Assignments are made on the basis of COSY, HSQC, HMBC and NOESY correlations, Chemical shift values are in δ (ppm), and Coupling constants (*J*) are in Hz

**Table 25** Experimental and literature ^13^C NMR data of compounds **6, 10, 11, 13** and **14**

| **P** | **^13^C δ 6 (CDCl_3_)** | | **^13^C δ 10(CDCl_3_)** | | **^13^C δ 11 (CDCl_3_)** | | **^13^C δ 13 (CDCl_3_)** | | **^13^C δ 14 (CDCl_3_)** | |
| --- | --- | --- | --- | --- | --- | --- | --- | --- | --- | --- |
| 1  2  3  4  5  6  7  8  9  10  11  12  1ʹ  2ʹ  3ʹ  4ʹ  5ʹ  6ʹ  7ʹ  8ʹ  2ʹʹ  3ʹʹ  2ʹʹʹ  1a  4a  6a  7a  8a  10a  11a  12a  OCH_3_ | **Expt.**  122.8  122.4  155.7  99.9  -------  66.9  104.7  141.8  148.2  93.8  -------  -------  -------  -------  -------  -------  -------  -------  -------  -------  145.1  106.2  101.3  116.5  153.5  40.6  117.9  -------  154.2  79.2  -------  ------- | **Lit.^7^**  122.9  122.4  155.7  99.9  -------  67.0  104.7  141.8  148.2  93.8  ------------------------------------------------------------------------  --------  145.1  106.3  101.3  116.5  153.5  40.6  117.9  -------  154.2  79.2  ---------------- | **Expt.**  -------  160.1  123.9  142.4  119.6  124.8  156.2  99.5  ------  ------  ------  ------  122.9  151.6  95.5  148.7  141.1  110.3  -------  -------  146.7  106.4  101.5  ------  116.2  ------  ------  157.8  -------  -------  -------  56.9 | **Lit.^7^**  -------  173.2  124.0  142.4  119.6  124.8  156.2  99.5  -------  -------  -------  -------  -------151.7  95.5  148.8  141.3  110.3  -------  -------  146.7  106.4  101.5  -------  116.2  --------  --------  156.2  --------  --------  --------  56.9 | **Expt.**  -------  71.3  48.33  192.7  120.9  115.5  159.2  99.7  -------  -------  -------  -------  122.6  152.7  95.4  147.8  141.3  109.8  -------  -------  146.0  107.1  101.3  ------  118.8  -------  -------  159.9  -------  -------  -------  56.5 | **Lit.^7^**  ------  71.3  48.3  192.6  120.8  115.6  159.2  99.6  --------  ---------  ---------  ---------  122.6  152.8  95.4  147.8  141.4  109.8  -------  -------  146.0  107.0  101.3  -------  118.8  -------  -------  159.9  ---------  ---------  ---------  56.5 | **Expt.**  106.8  142.3  149.5  99.2  -------  63.9  -------  100.0  158.3  123.3  121.0  192.9  -------  -------  -------  -------  -------  -------  -------  -------  146.5  105.7  101.3  109.2  149.6  75.9  160.3  -------  -------  114.6  68.3  ------- | **Lit.^7^**  106.8  142.3  149.5  99.9  -------  63.9  -------  100.0  158.3  123.3  121.0  192.9  -----------------------------------------------------------  ---------  ---------  146.4  105.7  101.3  109.2  149.6  75.9  160.3  -------  -------  114.6  68.3  ------- | **Expt.**  106.9  142.4  147.4  98.9  -------  66.4  -------  99.9  158.6  123.1  121.0  190.6  -------  -------  -------  -------  -------  -------  -------  -------  146.2  106.9  101.2  105.3  148.5  72.1  159.8  -------  -------  116.1  45.3  ------- | **Lit.^7^**  106.9  143.2  147.9  98.9  -------  66.4  -------  99.8  158.6  123.1  121.0  190.6  --------  --------  --------  --------  --------  --------  --------  --------  146.2  106.9  101.2  105.3  148.5  72.1  159.8  -------  -------  116.1  45.3  ------- |

**Table 26** Experimental and literature ^13^C NMR data of compounds **18, 20, 21** and **22**

| **P** | **^13^C δ 18 (CDCl_3_)** | | **^13^C δ 20 (CDCl_3_)** | | **^13^C δ 21 (CDCl_3_)** | | **^13^C δ 22 (CDCl_3_)** | |
| --- | --- | --- | --- | --- | --- | --- | --- | --- |
| 1  2  3  4  5  6  7  8  9  10  11  12  1ʹ  2ʹ  3ʹ  4ʹ  5ʹ  6ʹ  7ʹ  8ʹ  2ʹʹ  3ʹʹ  2ʹʹʹ  4ʹʹ  4a  4ʹʹʹ  5ʹʹʹ  8a  10a  11a  12a  OCH_3_ | **Expt.**  -------  66.5  39.4  78.3  132.3 109.7  160.2  103.6  156.7  112.6  -------  -------119.0  122.1  114.8  154.5  99.4  156.9  --------  --------76.8  127.6  --------121.9  --------28.0  27.8  -------  -------  -------  -------  ------- | **Lit.^14^**  -------  66.5  39.4  76.5  132.2  109.8  160.2  103.7  157.2  112.4  -------  -------  119.4  122.0  114.9  156.6  99.4  154.4  -------  -------  78.4  127.6  -------  122.1  -------  -------  26.9  -------  -------  -------  -------  ------- | **Expt.**  108.7  142.9  151.1 101.0  -------  63.8  -------  113.2  168.1  105.3  130.1  191.1  -------  -------  -------  31.1  87.9  144.0  112.7  17.1  -------  -------  -------  109.3  148.4  76.0  157.7  -------  -------  111.7  67.6  55.9  56.4 | **Lit.^15,16^**  108.8  142.9  151.2  101.1  -------  63.9  -------  113.2  168.0  105.3  130.2  191.1  --------  --------  --------  31.1  88.0  142.9  112.6  17.1  --------  --------  --------109.5  148.4  76.1  157.7  --------  --------111.8  67.6  55.9  56.4 | **Expt.**  110.4  157.9  167.4 100.9  -------  66.3  -------  112.9  149.5  104.9  130.0  188.9  --------  -------  -------  31.3  87.9  143.1  112.6  17.1  -------  -------  -------  104.8  143.9 72.2  147.4  -------  -------  113.4  44.6  55.9  56.3 | **Lit.^7,15^**  110.1  157.4  166.8  100.6  -------  65.9  -------  112.9  149.0  104.4  129.4  188.3  ----------  ----------  ----------  30.9  87.3  142.1  112.5  16.7  ----------  ----------  ----------  104.5  143.4  71.8  147.0  ----------  ----------  113.1  44.1  55.9  56.4 | **Expt.**  -------  154.7 121.1  176.6  119.0  126.0  157.3  99.8  -------  -------  -------  -------  112.9  153.0  95.5  148.5  141.3  111.3  -------  -------  147.4  107.1  101.4  -------  121.2  -------  -------  154.3  -------  -------  -------  56.9 | **Lit.^7^**  ------  154.7  121.1  176.6  119.0  126.0  157.2  99.8  -------  -------  -------  -------  112.8  153.0  95.5  148.4  141.2  113.3  -------  -------  147.4  107.0  101.4  -------  121.1  --------  --------  154.2  --------  --------  --------  56.9 |

**Table 27** Experimental and literature ^13^C NMR data of compounds **23** and **24**

| **P** | **^13^C δ 23 (CD_3_OD)** | | **^13^C δ 24 (CD_3_OD)** | |
| --- | --- | --- | --- | --- |
| 1  2  3  4  6  7  8  9  10  1a  4a  6a  7a  10a  11a  1'  2'  3'  4'  5'  6' | **Expt.**  131.8  113.9  154.1  103.8  66.3  124.6  107.3  160.5  97.3  112.9  154.9  39.6  118.1  158.4  78.5  30.1  77.0  69.2  19.6  24.5  ----- | **Lit.^17^**  133.3  115.4  155.6  105.3  67.7  126.0  108.7  159.9  98.8  114.4  156.4  41.1  119.5  162.0  79.9  31.6  70.6  78.4  21.0  26.0  ----- | **Expt.**  126.3  121.1  161.3  97.2  66.4  124.6  107.3  160.5  97.3  112.1  155.9  39.5  118.2  158.4  78.9  29.3  89.9  71.2  23.7  -----  23.9 | **Lit. ^17^**  127.7  122.5  162.7  98.6  67.8  126.0  108.7  158.8  98.8  113.6  157.4  40.9  119.0  162.0  80.4  30.8  91.4  72.5  25.2  -----  25.4 |

Data for compounds **5**-**24** (recorded as Compounds **1**-**20**) can be access online from the links below:

[**https://www.sciencedirect.com/science/article/pii/S003194222030488X?dgcid=author**](https://www.sciencedirect.com/science/article/pii/S003194222030488X?dgcid=author)

[**http://www.ccdc.cam.ac.uk/data_request/cif**](http://www.ccdc.cam.ac.uk/data_request/cif)

**References**

1. Lin, L. J., Huang, X. B., & Lv, Z. C. Isolation and identification of flavonoids components from *Pteris vittata* L. *SpringerPlus*. **2016**, *1*, 1649.
2. El-sawi, S. A., Sleem, A. A. Flavonoids and hepatoprotective activity of leaves of *Senna Surattensis* (Burm.f.) in CCl4 induced hepatotoxicity in rats. *Aust. J. Basic App. Sci.* **2010**, *6*, 1326-1334.
3. Koko, W. S., Mesaik, M. A., Ranjitt, R., Galal, M., Choudhary, M. I. Immunosuppressive phenolic compounds from *Hydnora abyssinica* A. Braun. *BMC Complement. Altern. Med*. **2015**, *15*, 400.
4. Kashiwada, Y., Iizuka, H., Yoshioka, K., Chen, R., Nonaka, G., Nishioka, I. Tannins and related compounds. XCIII. Occurrence of enantiomeric proanthocyanidins in the Leguminosae plants, *Cassia fistulan* L. and *Cassia javanica* L. *Chem*. *Pharm. Bull*. **1990**, *4*, 888-893.
5. Shi, T., Chen, H., Jing, L., Liu, X., Sun, X., Jiang, R. Development of a kilogram-scale synthesis of salidroside and its analogs. *Syn. Commun*. **2011**, *177*, 2594-2600.
6. Brink, A.J., Rall, G. J. H., Engelbrecht, J. Structures of some minor pterocarpans of *Neorautanenia edulis. Phytochemistry* **1974,** *8*, 1581-1585.
7. Puyvelde, L. V., Norbert, D., Kimpe, N. D., Jean‑Pierre, Mudaheranwa, J. P., Gasiga, A., Schamp, N., Jean‑Paul, Declercq, J. P., Meerssche, M. V. Isolation and structure elucidation of potentially insecticidal and acaricidal isoflovone‑ type compounds from *Neorautanenia mitis*. *J. Nat. Prod.* 1987, *50*, 349‑56.
8. Fraga, B. M., Gonzalez-Coloma, A., Alegre-Gomez, S., Lopez-Rodriguez, M., Amador, L. J., Daiz, C. E. Bioactive constituents from transformed root cultures of *Nepeta teydea*. *Phytochemistry* **2017,** *133*, 59-69.
9. Breytenbach, J. C., Rall, G. J. H. Structure and synthesis of isoflavonoid analogues from *Neorautanenia amboensis* Schinz. *J. Chem. Soc, Perkin Trans.* **1980,** 1804-1809.
10. Chaturvedula, V. S. P., Prakash, I. Isolation of Stigmasterol and β-Sitosterol from the dichloromethane extract of *Rubus suavissimus*. *ICPJ.* **2012,** *9*, 239-242.
11. Rall, G. J. H., Engelbreght, J. P., Brink, A. J. The chemistry of *Neorautanenia edulis* G.A, Sm. the constitution of (-)-2-isopentenyl-3-hydoxy-8-9 methylenedioxypterocarpan, a new pterocarpan from the root bark. *J. South Afri. Chem.* *Inst.* **1971,** *26*, 56-60.
12. Crombie, L., Whiting, D. A. The extractives of *Neorautanenia pseudopachyrrhiza* the isolation and structure of a new rotenoid and two isoflavanones. *J. Chem. Soc.* **1963,** 1569-1579.
13. Mitscher, L. A., Okwute, S. K., Gollapudi, S. R., Drake, S., Avona, E. Antimicrobial pterocarpans of Nigerian *Erythrina mildbraedii*. *Phytochemistry* **1988,** *11*, 3449-3452.
14. Nkengfack, A. E., Vardamides, J. C. Z., Tanee Fomum, Z. T, Meyer, M. Prenylated Isoflavanone from *Erythrina eriotricha*. *Phytochemistry* **1995,** *6*, 1803-1808.
15. Oberholzer, M. E., Rall, G. J. H., Roux, D. G., The concurrence of 12a-hydroxy- and 12a-*O*-methylrotenoids. Isolation of the first natural 12a-*O*-methylrotenoids. *Tetrahedron Lett.* **1974,** *25*, 2211-2214.
16. Phrutivorapongkul, A., Lipipun, V., Ruangrungsi, N., Watanabe, T., Ishikawa, T. Studies of the constituents of seeds of *Pachyrrhizus erosus* and their anti-herpes simplex virus (HSV) activities. *Chem. Pharm. Bull.* **2002,** *50*, 534-537.
17. Sakurai, Y., Sakurai, N., Masahiko, T., Nakanishi, Y., Bastow, K. F., Wang, X., Cragg, G. M., Lee, K. H. Rautandiols A and B, pterocarpans and cytotoxic constituents from *Neorautanenia mitis* *J. Nat. Prod.* 2006, *69*, 397‑399.
